# Tandem Mass Spectrometry Reflects Architectural Differences in Analogous, Bis-MPA-Based Linear Polymers, Hyperbranched Polymers, and Dendrimers

**DOI:** 10.1021/jasms.4c00330

**Published:** 2024-11-08

**Authors:** Kayla Williams-Pavlantos, McKenna J. Redding, Oluwapelumi O. Kareem, Mark A. Arnould, Scott M. Grayson, Chrys Wesdemiotis

**Affiliations:** † Department of Chemistry, University of Akron, Akron, Ohio 44325, United States; ‡ Department of Chemistry, Percival Stern Hall, Tulane University, New Orleans, Louisiana 70118, United States; § Bruker Daltonics LLC., 40 Manning Road, Billerica, Massachusetts 01821, United States

**Keywords:** tandem mass spectrometry, linear polymer, hyperbranched polymer, dendrimer, polyester

## Abstract

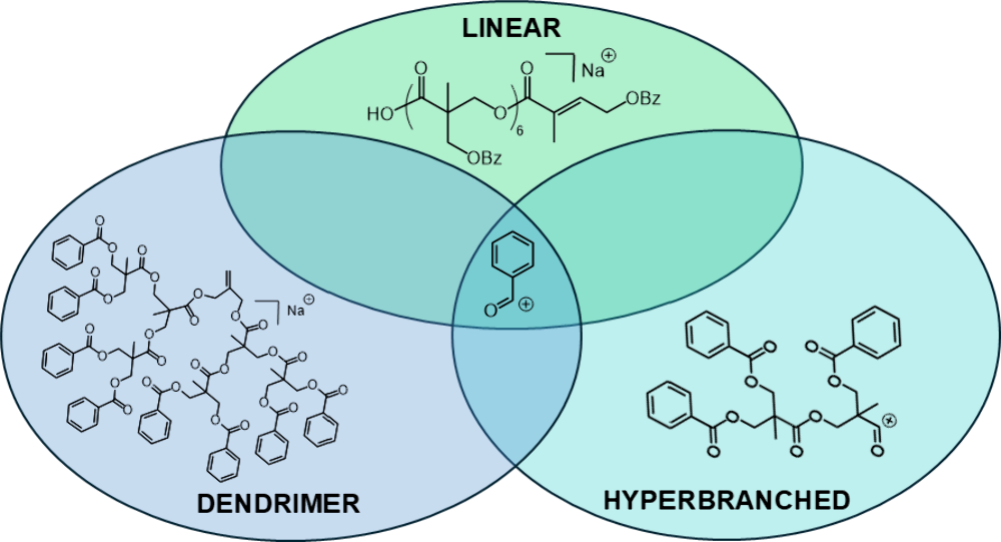

The growing use of branched polymers in various industrial and technological applications has prompted significant interest in understanding their properties, for which accurate structure determination is vital. This work is the first instance where the macromolecular structures of dendrimers, linear polymers, and hyperbranched polymers with analogous 2,2-bis(hydroxymethyl)propionic acid (bis-MPA) backbone groups were synthesized and analyzed via tandem mass spectrometry (MS/MS). When comparing the fragmentation pathways of these polymers, some unique and interesting patterns emerge that provide insight into the primary structures and architectures of each of these materials. As expected, the linear polymer undergoes multiple random backbone cleavages resulting in several fragment ion distributions that vary in size and end group composition. The hyperbranched polymer dissociates preferentially at branching sites; however, differently branched isomers exist for each oligomer size, thus giving rise again to several fragment distributions. In contrast, the dendrimer presents a unique fragmentation pattern comprising key fragment ions of high molecular weight; this unique characteristic stands out as a signature for identifying dendrimer structures. Overall, dendrimers, hyperbranched polymers, and linear polymers display individualized fragmentation behaviors, which are caused by differences in primary structure. As a result, tandem mass spectrometry fragmentation is a particularly useful analytical tool for distinguishing such macromolecular architectures.

## Introduction

Advances in soft ionization techniques over the last few decades have extended the applicability of mass spectrometry (MS) to larger macromolecules. The introduction of these techniques has made MS an effective method for synthetic polymer analysis.^1,2^ Single stage mass spectrometry separates ions by their unique mass-to-charge ratios (*m*/*z*), which can provide information about the purity of the sample and aid in end group and repeat unit identification.^2,3^ Although an accurate *m*/*z* value is relevant for the determination of elemental compositions, it does not directly reveal specific primary structure or sequence information.^2−6^ For this, tandem mass spectrometry (MS/MS) must be used, which exploits differences in bond stabilities to reveal insight about the connectivity and architecture of the parent structure.^2,3,7−10^ MS/MS has been effectively utilized for the identification of polymeric chain-end or in-chain substituents, the differentiation of isobaric and isomeric species, the determination of macromolecular architectures and sequences, and the identification of defective structures.^2,3,10−13^

### Effects of Branching

Structurally and chemically diverse polymers have been developed for a wide range of applications.^10^ Variation in sizes and compositions of polymers yield unique physical, chemical, and mechanical properties. For instance, longer linear polymers have more entanglement and higher melting points than shorter polymers of the same chemical composition.^14^ Another way to gain modular control over the properties of a polymer is by altering the connectivity of the monomers. For example, cyclic, star-shaped, comb-shaped, or other dendritic/branched structures can all be formed by tuning the synthetic process for specific bond connectivity.^10,15^

In order to keep up with the synthetic advances in polymer science, thorough structural characterization of the synthesized or modified polymers is required.^10^ Some of the major structural features that need to be identified by the employed characterization techniques include the type of functional groups, degree of branching (DB), and the connectivity of the monomer(s). Size exclusion chromatography (SEC) is useful for determining the molecular weight distribution and can give some insight into branching, as branched molecules exhibit reduced hydrodynamic volumes compared to their linear counterparts. However, a linear analogue is not always available for comparison which is required for complete branching characterization via SEC. If the branched and linear groups can be clearly distinguished by nuclear magnetic resonance (NMR) spectroscopy, the integrations can be used to determine the DB. The Frey equation, shown below, can be used to calculate the degree of branching based on the relative signals of the dendritic (*D*), linear (*L*), or terminal (*T*) monomer units.^16^
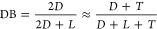


**Scheme 1:**
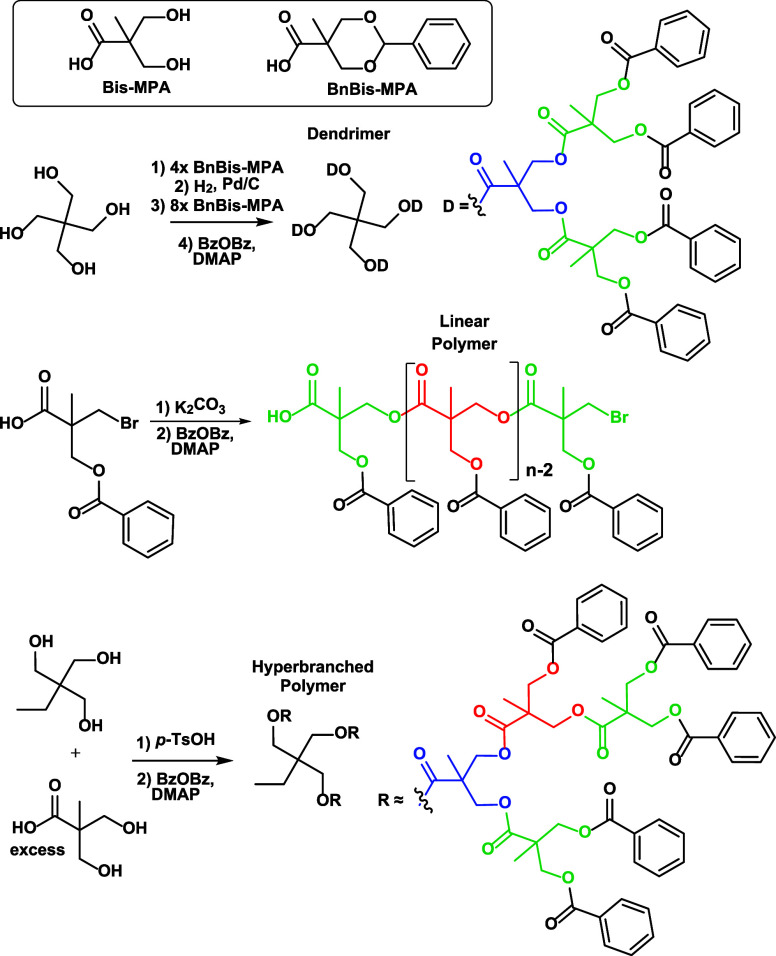
Polymers with the Bis-MPA Repeat Unit (C_5_H_7_O_3_, 115 Da) in Three Different Architectures The polymer repeat units are dendritic = blue, linear = red, and terminal = green; (a) the G2 dendrimer has perfect branching (blue in the core and green at the end groups), (b) the linear polymer has benzoyl groups at each position (all red except for the first and last green repeating units), and (c) the hyperbranched has random branching throughout (which will be blue and red in the middle and green at the end groups).

For large linear polymers, the DB is exactly 0.0 because there are no dendritic units, whereas for dendrimers, the DB is exactly 1.0 because there are no linear units.^16^ In this study, the 2,2-bis(hydroxymethyl)propionic acid (bis-MPA) repeat unit (Scheme 1) was used to instill the desired DB before benzoylation of the free hydroxy groups. While both the dendritic and linear monomers each contain one carboxylic acid group, the dendritic monomer consists of two free hydroxyl groups which can react further; meanwhile, the linear monomer consists of one free hydroxyl group and one protected benzoyl protected group.

Although NMR can be used to determine DB quantitatively, this is an average value of the entire sample. Additionally, NMR has limited sensitivity for differentiating short-chain from long-chain branching.^14^ On the other hand, mass spectrometry makes it possible to analyze complex polymeric samples and deduce composition and structural information based on individual oligomers, which are isolated from the bulk sample by their unique *m*/*z* ratio.

### Characterization of Dendrimers

Dendrimers are perfect, monodisperse, highly branched polymers.^17^ Since their discovery in the mid-1980s, Tomalia’s polyamidoamine (PAMAM) dendrimers have been the most widely accepted and extensively studied dendritic systems.^18,19^ They are usually synthesized via the divergent, or “bottom-up”, approach which, even under the most stringent preparation conditions for these amine-based dendrimers, generates impurities that cannot be avoided but can be identified and quantified.^15,17^ The characterization techniques developed over the last 40 years have greatly enhanced the understanding of the synthesis and impurities of such compounds.

Although this class of dendrimers has been heavily studied, it was not until 2012 that MS/MS was utilized for their characterization, thereby aiding in the discovery of some of the major impurities present in PAMAM dendrimers regardless of the core.^20^ This new knowledge has led to a variety of interesting and valuable dendritic systems and to the accessibility of new dendritic scaffolds.^17^

The monomer used in this study is 2,2-bis(hydroxymethyl)propionic acid (bis-MPA) which is classified an AB_2_-type monomer (Scheme 1).^21^ It is a relatively inexpensive and immensely versatile reagent. Similar to studies using PAMAM, a divergent approach was used in the synthesis of these dendrimers, but, unlike the PAMAM systems, highly pure bis-MPA based dendrimers can be synthesized in large quantities without the need for column chromatography.^22,23^

With the growing demand for dendritic and branched systems, the application of advanced analytical techniques have become increasingly important to fully understand their purity, chemical composition, morphology, shape, and homogeneity.^24,25^

### Architectural Comparisons via MS/MS

Polymerizations often do not allow for complete control of side product formation, which leads to impurities that are closely related to the intended product. Oftentimes, these complex mixtures can be difficult to characterize using single-stage MS methods.^2−6^ MS/MS, on the other hand, can provide structural identification, such as individual end group composition, sequence, and architecture assignment. This capability is especially beneficial for the differentiation of isobaric and isomeric species as well as linear vs nonlinear systems.^10^ Analogous linear and branched systems can either produce distinct fragment ions or the same fragment ions with varying intensities based on the degree of branching and length of the branches.^8,26^ For example, a dramatically different fragmentation pattern is observed for an in-chain-substituted linear polystyrene compared to a four-arm star-branched polystyrene.^3^ Another method of identifying the presence of branched architecture(s) is by detecting fragments that were impossible to form by isomeric linear structures.^27,28^ An example of this comes from Chaicharoen et al., who used strategically placed ester groups for the characterization of polyacrylates. The esters were adjacent to the branching points, which is where bond cleavages occurred, leading to fragment ions that were characteristic of the location and size of the branches.^27^ From such product ions, precursor ion structures can be reconstructed, and fragmentation mechanisms can be established. Understanding the fragmentation mechanism enhances the confidence in the structural assignment of the precursor ion, which is especially important when analyzing mixtures or blends.^8^

There have been many studies using MS/MS to analyze polymers but not as many that compare polymer architectures. The architectural studies that have been published compared linear polymers to cyclic polymers,^3,6−8,29,30^ hyperbranched polymers to their linear analogues,^27,29,31^ and star polymers and their linear counterparts,^14,26^ but none of them compared all three branched architectures, viz., a linear polymer, a hyperbranched polymer, and a dendrimer. One of the reasons for this has been the synthetic inaccessibility of bis-MPA linear analogues; in contrast, the dendritic and hyperbranched structures can, generally, be produced more easily.^17,32^ Herein, we describe the first MS/MS comparison of a dendrimer, hyperbranched polymer, and linear polymer with analogous bis-MPA repeating units and benzoyl ester terminal functional groups.

## Experimental Section

### Characterization

The three architectures studied in this work are a dendrimer (Schemes 1a and S1; Figures S1 and S2), a linear polymer (Scheme 1b, Figures S3 and S4), and a hyperbranched polymer (Schemes 1c and S2; Figures S5–S7). NMR spectra were measured on a Bruker AVANCE 300 MHz spectrometer (Figures S2, S4, S5, and S7). Proton (^1^H) NMR (300 MHz) experiments were performed at ambient probe temperature at a concentration of 12–50 mg/mL in chloroform-*d* (CDCl_3_) or methanol-*d* (CD_3_OD), purchased from Cambridge Isotope Laboratories (Andover, MA). An appropriate number of scans were used for each sample to obtain a significant signal-to-noise ratio with a relaxation delay of 2–5 s depending on the sample.

**Figure 1:**
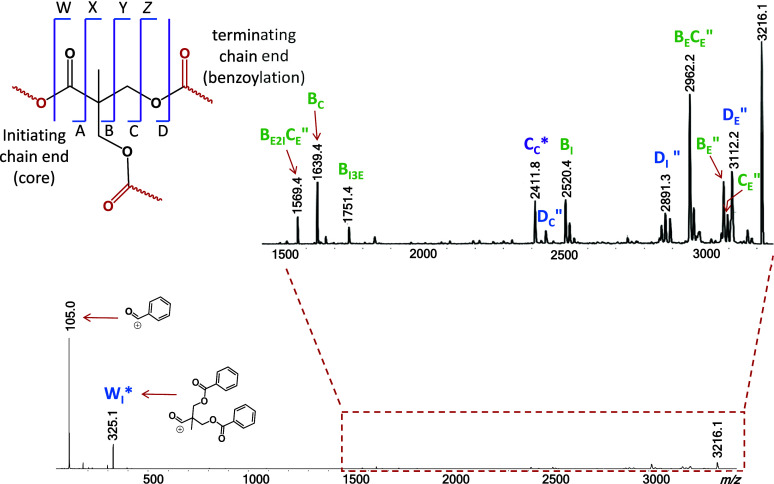
MALDI-ToF MS/MS spectrum of sodiated tetra[G2]Bz_16_ (*m*/*z* 3216.1). The fragment nomenclature is explained in the top-left inset (see also text). The subscripts indicate whether fragmentation took place at/near the core (C), at an interior site (I), or at an exterior site (E); consecutive fragmentations are indicated in the subscript. For example, B_I3E_ arises after bond dissociations at an interior site followed by three losses at an exterior (periphery) site; a double prime indicates fragments with a new saturated chain end, otherwise dissociation generated a terminal alkene. An asterisk indicates fragment ions without Na^+^. See schemes for mechanistic rationalizations.

A Bruker Autoflex III matrix-assisted laser desorption/ionization time-of-flight mass spectrometer (MALDI-ToF MS) (Bruker Daltonics, Billerica, MA) was used in the initial characterization of samples to ensure reaction completion (Figures S1a,b, S3a,b, S6a). A Bruker UltraFlex III MALDI-ToF/ToF mass spectrometer (Bruker Daltonics, Billerica, MA) equipped with a LIFT cell was used to acquire the MS/MS spectra with no additional collision gas (Figures 1, 3, and 6); the precursor ion isolation window in LIFT mode is ∼5 *m*/*z* units.^9,26,30,33^ All mass spectra were collected in positive reflectron ion detection mode. Typical preparation for all samples included the use of a stock matrix solution (60 mg/mL in tetrahydrofuran (THF)), analyte (10 mg/mL in THF), and a stock cation solution (5 mg/mL in THF). The stock solutions were combined in a 20:1:1 μL ratio (v/v/v) (matrix/analyte/cation) and plated via the dried droplet method. *trans*-2-[3-(4-*tert*-Butylphenyl)-2-methyl-2-propenylidene]malononitrile (DCTB) from TCI America (Portland, OR) was used as the matrix for all samples. Sodium (Na^+^) trifluoroacetate was used as the cationization salt for all samples. SpheriCal dendrimers (Polymer Factory, Stockholm, Sweden) were used as mass spectrometry calibration standards. All quoted masses and *m*/*z* data correspond to monoisotopic values; the *m*/*z* data in figures and schemes are measured values.

Gel permeation chromatography (GPC) analysis was performed on a Waters model 1515 equipped with an isocratic pump and a Waters model 2414 differential refractometer detector (Waters Corp., Milford, MA), using a Polymer Service Standards (PSS) SDV analytical 100 Å 3 mm (8 × 300 mm) guard column and three PSS SDV analytical linear M 3 mm (8 × 300 mm) columns in series (Polymer Laboratories Inc., Amherst, MA) (Figures S1c, S3c, and S6b). Data were collected in THF at a flow rate of 1 mL/min at 30 °C; *M*_n_, *M*_w_, and *Đ* values were calibrated against Polystyrene ReadyCal Standards from Waters.

### Synthesis of the Pentaerythritol-Core Second-Generation Deprotected Dendrimer (Tetra[G2]OH_16_)

Tetra[G2]OH_16_ was synthesized using previously published experimental conditions (cf. Schemes 1a and S1).^34^

### Benzoyl (Bz) Functionalization of the Dendrimer

A round-bottomed flask was charged with a stir bar and placed in a sand bath heated to 55 °C. Chloroform (CHCl_3_) (50 mL), 4-dimethylaminopyridine (DMAP) (0.19 g, 1.57 mmol), and benzoic anhydride (0.78 g, 3.47 mmol) were added to the round-bottom flask, followed by the deprotected starting material, tetra[G2]OH_16_ (0.17 g, 0.11 mmol). The reaction was left to stir for 16 h under inert atmosphere (N_2_). A crude MALDI-ToF MS spectrum was obtained to ensure the complete functionalization of the respective starting material. The reaction was quenched with deionized water (DI H_2_O) (100 mL) for 1 h. Next, the (DI H_2_O) was replaced with a saturated potassium carbonate (K_2_CO_3_) solution (100 mL), which was left to stir for 16 h to ensure the complete removal of excess anhydride and acid. The CHCl_3_/K_2_CO_3_ solution was rinsed into a separatory funnel using CHCl_3_. The organic layer was washed three times with sodium bicarbonate (NaHCO_3_), three times with sodium bisulfate (NaHSO_3_), and then once with DI H_2_O. The organic layer was collected, dried over magnesium sulfate (MgSO_4_), and then filtered. CHCl_3_ was evaporated via rotary evaporator, and the resulting white solid (tetra[G2]Bz_16_) was further dried under high vacuum: (0.30 g, 85.0%) MALDI-ToF MS (Figure S1a-b) *m*/*z* 3216.21. GPC (Figure S1c) *M*_n_: 2,400 *Đ*: 1.01. ^1^H NMR (Figure S2) (CDCl_3_, 300 MHz): δ 1.17 (s, 12H, CH_3_), 1.33 (s, 24H, CH_3_), 4.11 (s, 8H, CH_2_), 4.26 (d, *J* = 11.1 Hz, 8H, CH_2_), 4.36 (d, *J* = 11.1 Hz, 8H, CH_2_), 4.48 (d, *J* = 11.2 Hz, 16H, CH_2_), 4.55 (d, *J* = 11.2 Hz, 16H, CH_2_), 7.37 (t, *J* = 7.5 Hz, 32H, CH _*m*-Ar–H_), 7.49 (t, *J* = 7.5, 16H, CH _*p*-Ar–H_), 7.95 (d, *J* = 7.5 Hz, 32H, CH _*o*-Ar–H_).

### Poly(3-(benzoyloxy)-2-(bromomethyl)-2-methylpropanoic acid) (PBBM)

The linear polymer was synthesized according to previously described methods:^34^ (0.74 g, 73.8%) MALDI-ToF MS (Figure S3a,b) *M*_n_: 2,600 *Đ*: 1.06; GPC (Figure S3c) *M*_n_: 1,300 *Đ*: 1.09; ^1^H NMR (Figure S4) (CDCl_3_, 300 MHz): δ 1.27 (s, 30H, CH_3 backbone_), 1.36 (s, 3H, CH_3 end group_), 3.64 (m, 2H, CH_2_Br), 4.39 (20H, CH_2 backbone_), 7.35 (m, 20H, CH_*m*-Ar–H_), 7.52 (m, 10H, CH_*p*-Ar–H_), 7.92 (m, 20H, CH_*o*-Ar–H_).

### Polycondensation of Bis-MPA and Trimethylolpropane (TMP)^35^

A predried three-neck round-bottomed flask was fitted with a drying column on the first neck, N_2_ through an adapter on the second neck, and a sealed mechanical stirrer on the third neck. The round-bottom was placed in an oil bath preheated to 140 °C. TMP (0.74 g, 5.52 mmol) and *p*-toluene sulfonic acid (*p*-TsOH, 0.14 g, 0.79 mmol) were added to the round-bottom flask, followed by the addition of bis-MPA (2.12 g, 15.88 mmol). The reaction was left to stir under inert atmosphere (N_2_). After 0.5, 1, and 1.5 h, additional bis-MPA (2.12 g, 15.88 mmol) was added. After 2 h, *p*-TsOH (0.27 g, 1.57 mmol) and bis-MPA (2.12 g, 15.88 mmol) were added. Three additional bis-MPA aliquots (2.12 g, 15.88 mmol) were added 0.5 h apart until all eight aliquots were been added, and then the reaction was left to stir for up to 10 h or until the reaction solidified. Aliquots were removed from the reaction at various times to yield a range of molecular weights. The reaction mixture was dissolved in tetrahydrofuran (THF), and then a rotary evaporator was used to yield either an orange viscous material or an orange cloudy gel (∼12 g, ∼66%), cf. Schemes 1c and S2. ^1^H NMR (Figure S5) (CD_3_OD, 300 MHz): δ 0.94 (m, 3H, CH_3 core_), 1.16 (s, 27H, CH_3 terminal_), 1.22 (s, 57H, CH_3 linear_), 1.32 (s, 24H, CH_3 dendritic_), 3.69 (m, 94H, CH_2 core+backbone_), 4.26 (m, 58H, CH_2 backbone_)

### Benzoyl Functionalization of TMP-Initiated Hyperbranched Polymer

A round-bottomed flask was charged with a stir bar and placed in a sand bath heated to 55 °C. CHCl_3_ (100 mL), DMAP (0.89 g, 7.27 mmol), and benzoic anhydride (10.35 g, 45.73 mmol) were added to the round-bottom flask, followed by the hyperbranched polymer (1.03 g, 0.26 mmol). The reaction was left to stir for 72 h under inert atmosphere (N_2_). A crude MALDI-ToF MS spectrum was obtained to ensure the complete functionalization of the starting material. The reaction was quenched with DI H_2_O (200 mL) for 1 h. Next, the DI H_2_O was replaced with a saturated K_2_CO_3_ solution (200 mL), which was left to stir for 16 h to ensure the complete removal of excess anhydride and acid. The CHCl_3_/K_2_CO_3_ solution was rinsed into a separatory funnel using CHCl_3_. The organic layer was washed three times with NaHCO_3_, three times with NaHSO_3_, and then once with DI H_2_O. The organic layer was collected, dried over MgSO_4_, and then filtered. CHCl_3_ was evaporated via a rotary evaporator and the resulting white solid was further dried under high vacuum (1.65 g, 82.9%), cf. Scheme S2.

### Fractional Precipitation of Hyperbranched Polymer

The polymers were characterized by GPC, and the dispersity of all the samples was determined to be above 2. To reduce the dispersity to 1.1 or below, fractional precipitation was used. The samples were fully dissolved in a minimal amount of CHCl_3_. Diethyl ether (DEE) was slowly added while the sample was stirred until a permanently cloudy solution was obtained. The solution was filtered through a Whatman filter, and then the separated product was washed through with additional CHCl_3_. This process was repeated multiple times; each fraction was analyzed via GPC and reprecipitated as needed to achieve a dispersity of 1.1 or lower: (0.20 g, 10.1%) MALDI-ToF MS (Figure S6a) *M*_n_: 2800 *Đ*: 1.05. GPC (Figure S6b) *M*_n_: 2,400 *Đ*: 1.05. ^1^H NMR (Figure S7) (CDCl_3_, 300 MHz): δ 0.90 (t, 3H, CH_3 core_), 1.15–1.42 (bs, 30H, CH_3_), 3.84–4.69 (m, 48H, CH_2 core and backbone_), 7.37 (m, 26H, CH_*m*-Ar–H_), 7.54 (m, 13H, CH_*p*-Ar–H_), 7.94 (m, 26H CH_*o*-Ar–H_).

## Results and Discussion

For the bis-MPA monomer, the methyl group on each bis-MPA unit can be used to determine if the unit is branched or linear via NMR. Since the dendrimer, hyperbranched polymer, and linear polymer contain the same bis-MPA based monomer, the chemical shifts can be instrumental in labeling the units as branched or linear. The G2 dendrimer has four internal dendritic units (∼1.35 ppm), eight terminal benzoyl functionalized units (∼1.22 ppm), and no linear units, which gives it a DB of 1.0 (Figure S2). The linear polymer has benzoyl-functionalized linear units ranging from 1.16 to 1.32 ppm, with the brominated end group at ∼1.38 ppm, and no dendritic units, yielding a DB of 0.0 (Figure S4).

Malmström et al. first characterized their hyperbranched polymers using NMR spectroscopy; however, these had hydroxyl termination rather than benzoyl groups. They found that in acetone-*d*_6_ the dendritic units are observed at about 1.28 ppm, the linear units are detected around 1.18 ppm, and the terminal units are seen at approximately 1.06 ppm.^35^ In this study, the hydroxy-terminated hyperbranched polymer, which is the precursor for the benzoyl-functionalized hyperbranched polymer, was used to determine the degree of branching before esterification. In methanol-*d*_4_, the hyperbranched polymer was shown to have an average of 8 dendritic units at about 1.32 ppm, 19 linear units around 1.22 ppm, and 9 terminal units at approximately 1.16 ppm, yielding a DB of roughly 0.5 (Figure S5).

In order to investigate the various fragmentation pathways through which the different architectures proceed, the dendrimer, hyperbranched polymer, and linear polymer (all benzoyl functionalized) were subjected to MS/MS fragmentation. The fragmentation pathways of similar molecular weights were compared to differentiate the architecture-based fragmentation behavior.

### MS/MS of the Benzoylated Dendrimer

The MS spectrum of the dendrimer contains only two ions, the major one corresponding to the completely functionalized sodiated dendrimer and the minor one to the same dendrimer cationized with a potassium ion (Figure S1). The sodiated tetra[G2]Bz_16_ (*m*/*z* 3216.1) was selected for fragmentation (cf. Figure 1).

**Scheme 2:**
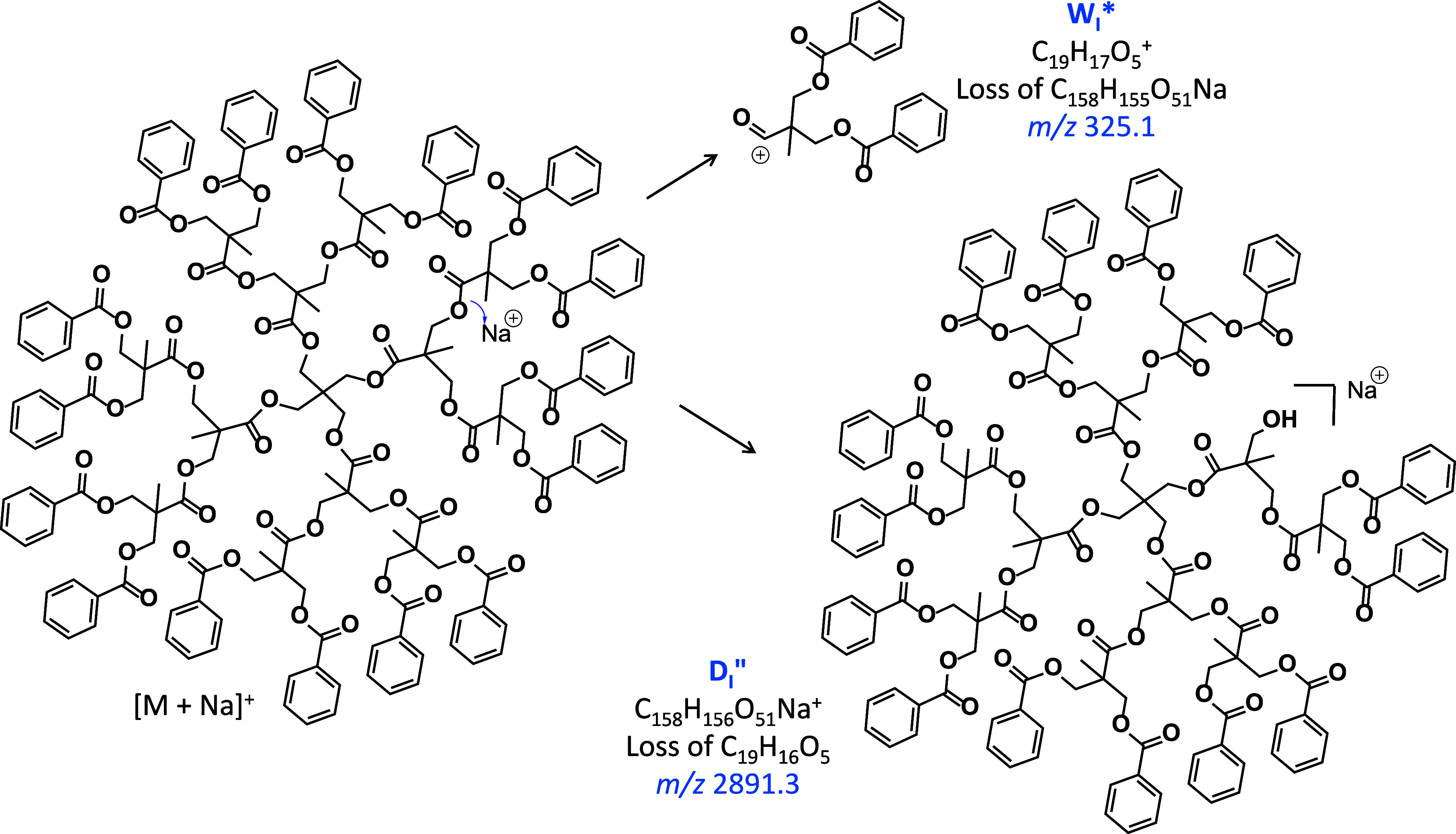
Charge-Induced Bond Cleavage of an Interior Ester Bond in the Sodiated Dendrimer, Leading to (top right) the Acylium Ion W_I_* via Loss of a Sodium Alkoxylate or (bottom right) after Proton Abstraction from the Acylium Ion by the Alkoxylate (cf. Scheme S3), to a Truncated Dendrimer with a Primary OH Group at an Interior Site (fragment D_I_″)

The major fragment ions from the sodiated dendrimer are generated by ester bond cleavages at exterior and interior sites, which give rise to the benzoyl cation (C_7_H_5_O^+^ at *m*/*z* 105.0) and an acylium cation composed of one doubly benzoylated bis-MPA unit (C_19_H_17_O_5_^+^ at *m*/*z* 325.1, W_I_* ion), respectively. The complementary fragments, viz. those produced by losses of C_7_H_4_O (104.0 Da) and C_19_H_16_O_5_ (324.1 Da) are also observed at *m*/*z* 3112.2 (D_E_″) and 2891.3 (D_I_″), respectively. These fragments are attributed to charge-induced dissociations promoted by the Na^+^ Lewis acid, as rationalized in Schemes 2 and S3 for the formation for W_I_* and D_I_″.

Analogous charge-induced dissociation at the core ester functionalities is also observed, leading to fragment D_C_″ (*m*/*z* 2451.8); its structure (together with that of fragment D_E_″) is shown in Figure S8. The complementary acylium cation for D_C_″ (i.e., W_C_* at *m*/*z* 765.3) is not detected above noise level, presumably because of facile consecutive fragmentation. Since the number of ester groups increases from the core (4) to the interior or first generation (8) and further to the exterior or second generation (16) sites, the probability of charge-induced fragmentation should also increase in this direction, as indeed affirmed by the relative abundance order D_C_″ < D_I_″ < D_E_″, cf. Figure 1.

**Scheme 3:**
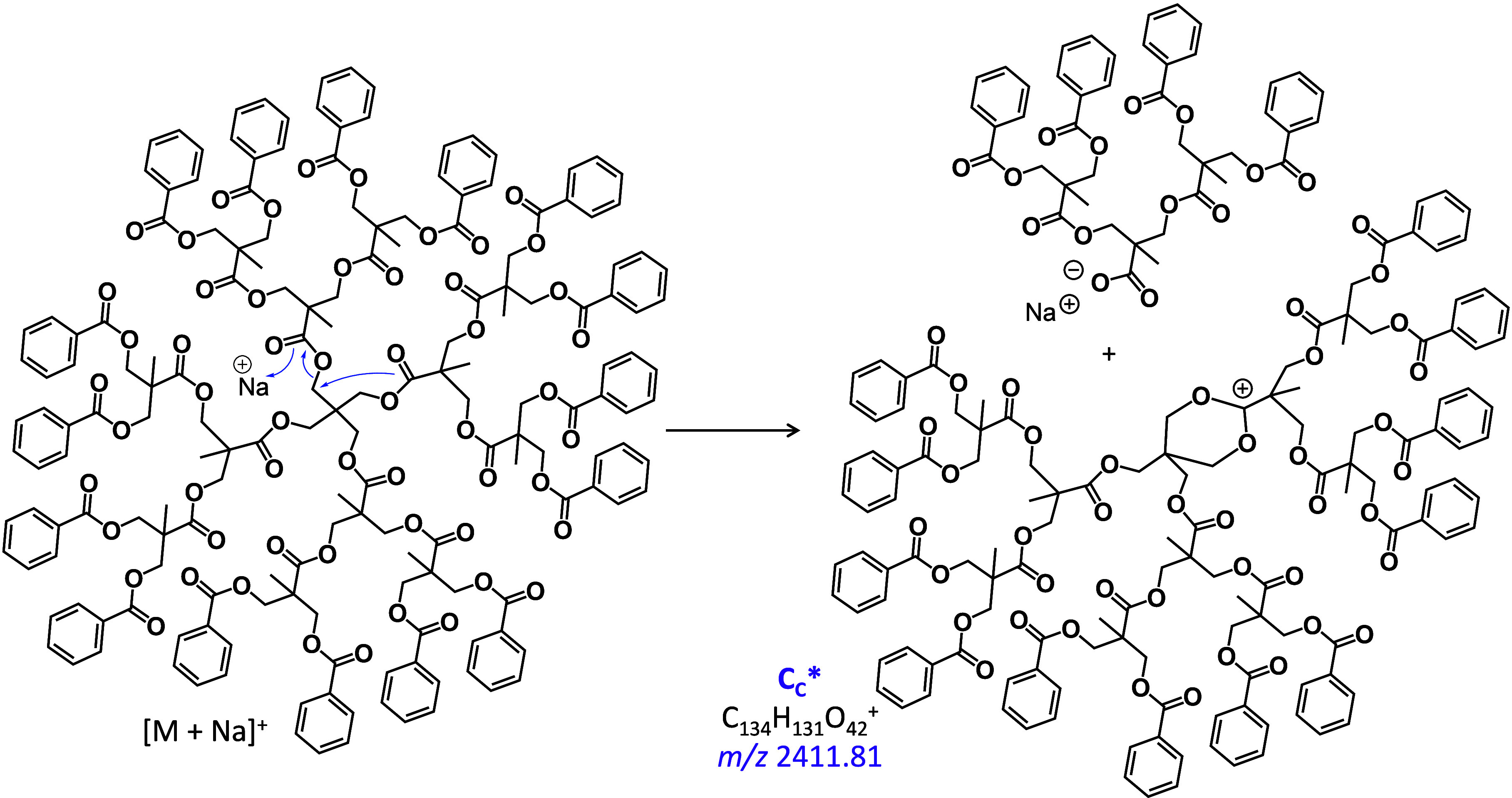
Charge-Induced Bond Cleavage of a Core COO–CH_2_ Bond in the Sodiated Dendrimer, Leading to Fragment C_C_* with a Dioxanylium Structure by Elimination of a Sodium Carboxylate

A different type of charge-induced fragmentation, involving the COO–CH_2_ instead of the CO–O ester bonds at the core, is invoked to justify the unique fragment at *m*/*z* 2411.8 (C_C_*). This dissociation is proposed to proceed via heterolytic COO–CH_2_ bond cleavage, facilitated by Na^+^, and concomitant intramolecular cyclization to form a six-membered ring dioxanylium cation by release of a neutral sodium carboxylate fragment (cf. Scheme 3). Such intramolecular nucleophilic substitution (S_N_i) reactions have been observed in the chemical ionization spectra of diesterified 1,3-diols.^36^

**Figure 2:**
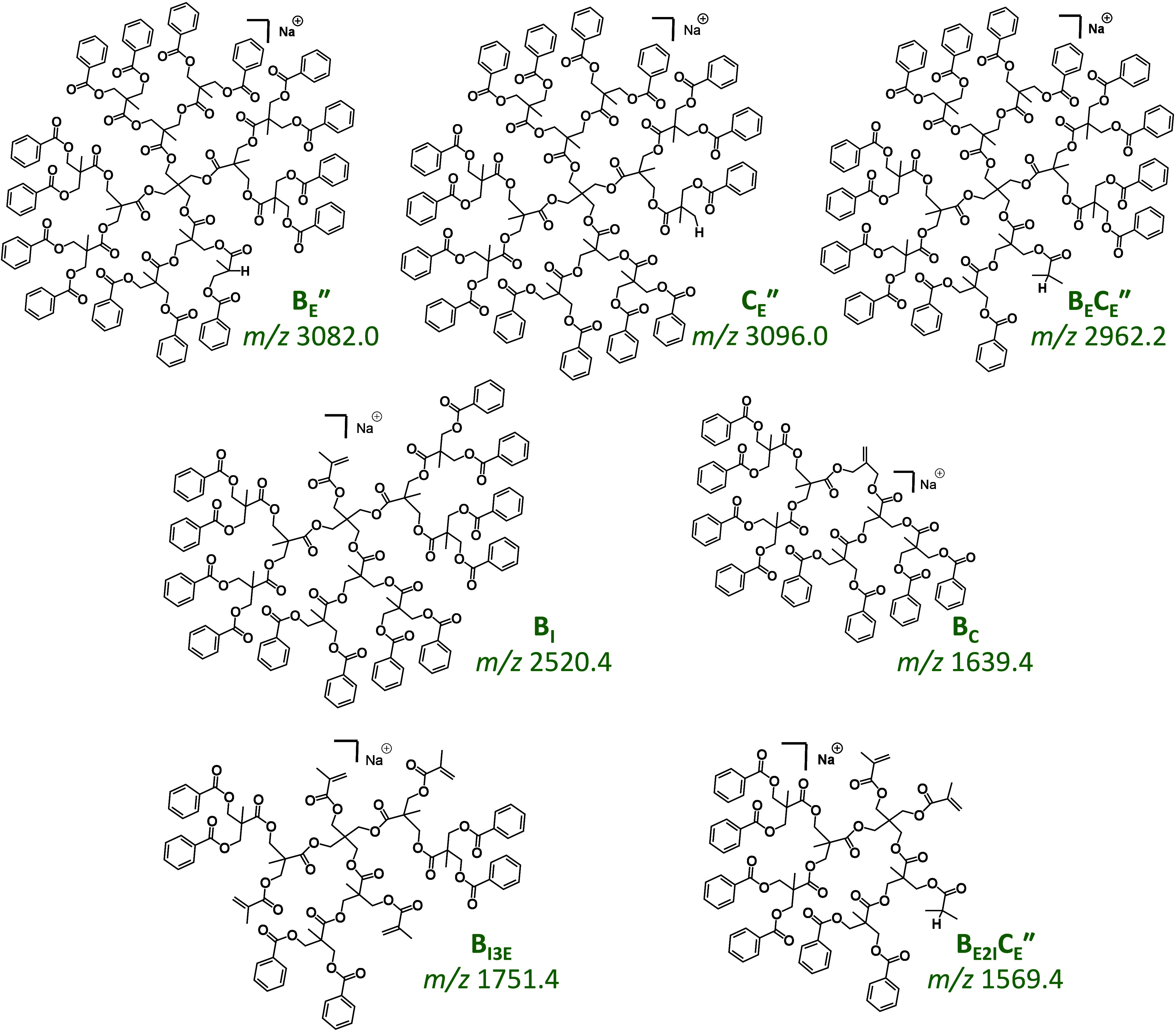
MS/MS fragments from sodiated tetra[G2]Bz_16_, formed via charge-remote dissociation pathways, as depicted in Schemes S4–S8. B_E_″, C_E_″, and B_E_C_E_″ (top): fragments formed by homolytic bond cleavage accompanied by H-rearrangement at the benzoylated bis-MPA repeating units, cf. Schemes S4, S5, and S6, respectively. B_I_ and B_C_ (center): fragments formed by homolytic bond cleavage followed by β scission in the newly created radical, yielding unsaturated terminal esters with an isopropenyl or isobutenyl substituent, respectively, cf. Schemes S7 and S8. B_I3E_ (bottom left): fragment formed by consecutive dissociation of B_I_ via elimination of three exterior branches following a pathway similar to that shown in Scheme S7. B_E2I_C_E_″ (bottom right): fragment formed by consecutive dissociation of B_E_C_E_″ after elimination of two interior branches according to the mechanism in Scheme S7.

Polyester [M + Na]^+^ ions have been shown to fragment via both charge-induced as well as charge-remote pathways.^8^ Dissociations via the latter mechanism can explain the remaining fragments at *m*/*z* > 1500 from sodiated tetra[G2]Bz_16_. The fragments at *m*/*z* 3096.0 (C_E_″), 3082.0 (B_E_″), and 2962.2 (B_E_C_E_″) correspond to neutral losses of 120 Da (C_7_H_4_O_2_), 134 Da (C_8_H_6_O_2_), and 254 (= 120 + 134) Da, respectively, which are most likely eliminated from the benzoylated peripheral repeat units (the notation B_E_C_E_″ indicates consecutive fragmentation of 134 Da from B_E_″ or 120 Da from C_E_″). Charge-remote homolytic bond cleavages accompanied by H-rearrangement and lactone formation in the neutral species being eliminated can account for these fragments, as depicted in Schemes S4–S6; the resulting fragment structures are also shown in Figure 2 (top) for clarity.

The fragmentation mechanism changes for B_I_ and B_C_, which arise by bond dissociations at the interior and core positions of the dendrimer that carry no benzoyl substituents. These fragments are reconciled by charge-remote homolytic C–C bond cleavage to produce radical intermediates, which subsequently decompose via β C–O bond scission to yield truncated dendrimers with an alkene end group, as illustrated in Schemes S7 and S8 for the formation of B_I_ and B_C_, respectively; the structures of these fragments are included in Figure 2 (center).

Finally, fragment ions B_I3E_ (*m*/*z* 1751.4) and B_E2I_C_E_″ (*m*/*z* 1569.4), shown at the bottom of Figure 2, can be explained by consecutive fragmentation of B_I_ and B_E_C_E_″, respectively, via homolytic bond cleavages and radical intermediates according to the mechanism shown in Scheme S7 for the B_I_ fragment. Specifically, B_E2I_C_E_″ can be formed from B_E_C_E_″ by elimination of two interior branches (denoted by the subscripted 2I), each removing a C_39_H_36_O_12_ (696.2 Da) segment from the dendrimer, while B_I3E_ can be formed from B_I_ by elimination of three exterior branches (denoted by the subscripted 3E), each removing a C_15_H_12_O_4_ (256.1 Da) segment from the dendrimer. These dissociations lead to unsaturated isopropenyl end groups at the truncation sites (cf. Figure 2, bottom). Interestingly, a distribution of fragments from elimination of more or less analogous branches is not observed, which is a unique characteristic of the dendrimer architecture, as will be evident from the comparison with linear and hyperbranched architectures (vide infra).

### MS/MS of the Linear Polymer PBBM

**Figure 3:**
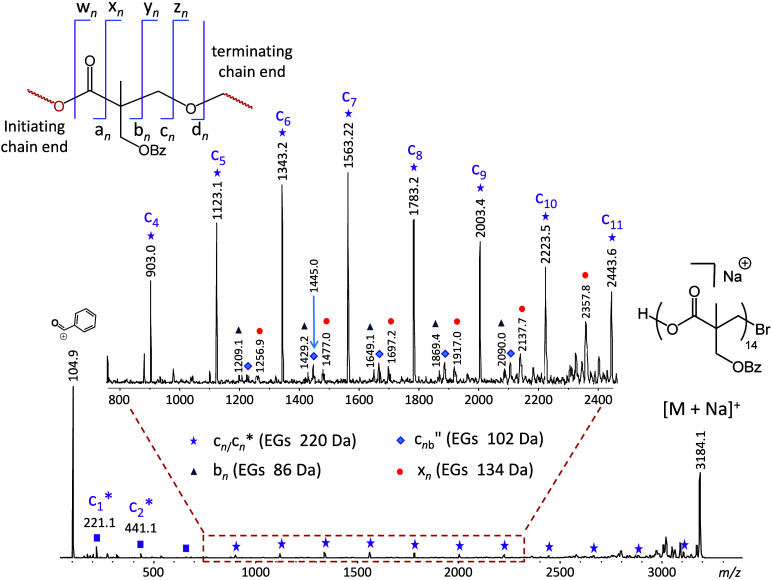
MALDI-ToF MS/MS spectrum of the sodiated PBBM 14-mer, Br-(C_12_H_12_O_4_)_14_-H (*m*/*z* 3184.1). The fragment nomenclature is explained in the top-left inset; low-case acronyms are used to distinguish fragments from linear vs branched architectures. The subscripts indicate the number of complete or partial repeat units in the fragment. A double prime indicates fragments with a new saturated chain end, while an asterisk indicates fragment without Na^+^. The mass of the fragment end groups (EGs) is given in parentheses next to the fragment acronym. See schemes for mechanistic rationalizations.

The synthesized linear PBBM chain has the primary structure Br-(C_12_H_12_O_4_)-H, where C_12_H_12_O_4_ (220.1 Da) is the repeat unit of this polymer (cf. Scheme 1b and Figure S3). For a meaningful architectural comparison, a linear *n*-mer with comparable mass to that of the previously studied dendrimer should be chosen. This stipulation was best fulfilled with the PBBM 14-mer whose [M + Na]^+^ ion (*m*/*z* 3184.1) and the [M + Na]^+^ ion of the dendrimer (*m*/*z* 3216.1) have similar masses. In similarity with the dendrimer, the most abundant fragment in the MS/MS spectrum of the sodiated PBBM 14-mer is the benzoyl cation (C_7_H_5_O^+^), cf. Figure 3; this ion can be formed by charge induced, heterolytic PhCO–O bond cleavage at any of the 14 side chains. It is noteworthy that an ion of *m*/*z* 325.1, which was abundantly present in the MS/MS spectrum of the dendrimer (Figure 1), is barely above noise level for the linear polymer (cf. Figure S9), consistent with the absence of doubly benzoylated bis-MPA units in the linear architecture.

The fragments in the high mass region of the MS/MS spectrum (*m*/*z* > 3000) are attributed to small neutral losses from the side chains and end groups. Likely structures of the eliminated molecules or radicals are provided in the zoomed view of this spectral region in Figure S10.

**Scheme 4:**
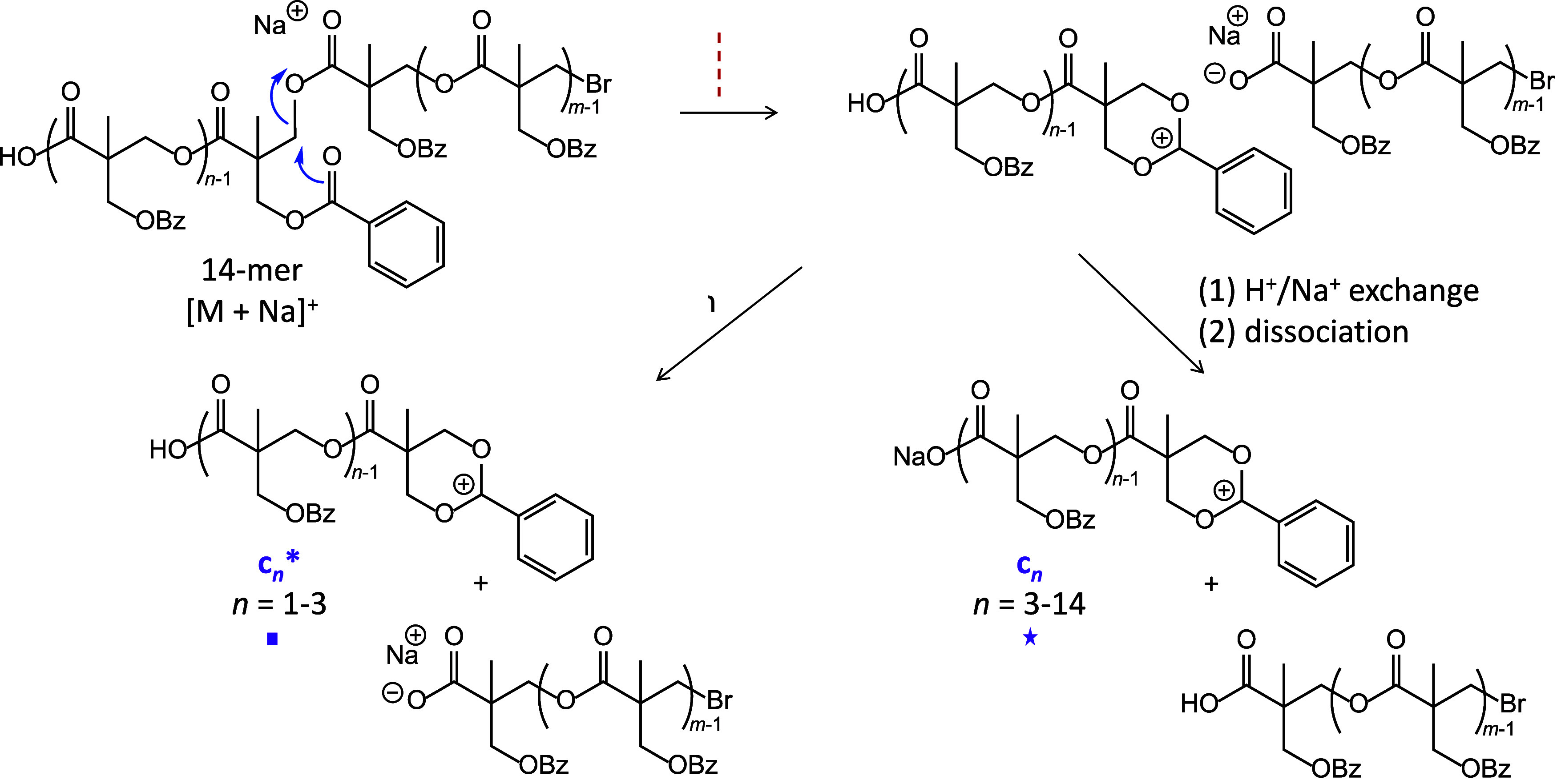
Charge-Induced Bond Cleavage of a CH_2_–OCO Bond in the Sodiated PBBM 14-mer, Leading to Fragment Ions Having the Overall Composition [(C_12_H_12_O_4_)_*n*_ + H]^+^ (c_n_*) or [(C_12_H_12_O_4_)_*n*_ + Na]^+^ (c_*n*_) Note that c_14_ is formed by cleavage of the CH_2_–Br bond and elimination of HBr.

Unlike the dendrimer, the linear polymer dissociates to produce a distribution of fragments composed of *n* repeat units, (C_12_H_12_O_4_)_*n*_, and no nominal end groups which is equivalent of having combined end groups with the same composition and mass as one repeat unit (i.e., C_12_H_12_O_4_, 220.1 Da). These fragments are observed as sodium-free ions (labeled by a purple square and c_n_* in Figure 3), or as sodiated species (labeled by a purple star and c_*n*_) and form a contiguous series extending from the monomer (c_1_*) to the 14-mer (c_14_). This fragmentation pattern can be rationalized by charge-induced cleavages at the CH_2_–OCO bonds (at the CH_2_–Br bond for c_14_), involving nucleophilic displacement of a carboxylate (bromide for c_14_) by the benzoyl group on the side chain, as illustrated in Scheme 4.^36^ The fragment ions resulting in this process contain a newly created dioxanylium chain end and a free COOH substituent at the other chain end, while the eliminated neutral chain segments have sodium carboxylate and bromine end groups (NaBr for c_14_). Noncovalent interactions between these incipient fragments permits H^+^/Na^+^ exchange before dissociation to the observed c_n_* and c_*n*_ series (cf. Scheme 4). The sodiated fragments (c_*n*_) dominate above three repeat units, as their longer chains can more easily provide multidentate coordination to Na^+^. Meanwhile, the inclusion of a benzoyl substituent on each PBBM monomer enables such a fragmentation pathway at each repeat unit, justifying the observation of a contiguous c_n_*/c_*n*_ fragment series across the entire chain length.

**Figure 4:**
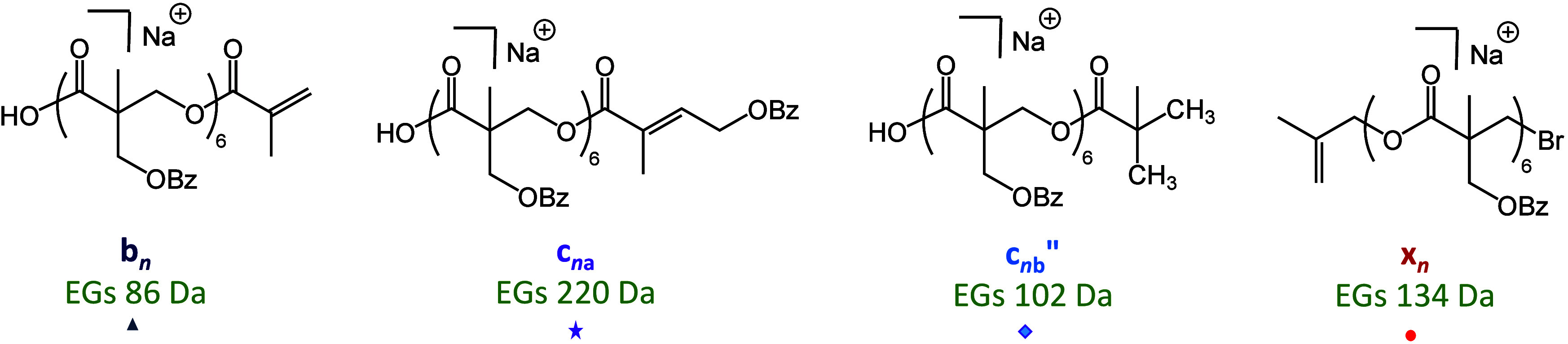
MS/MS fragments from sodiated PBBM, formed via charge-remote dissociation pathways involving hydrogen atom or alkyl group rearrangement(s) and β bond scissions, as rationalized in Schemes S9–S11. The total mass of the corresponding end groups (EGs) is given under the acronym (see Figure 3, top left, for the fragment nomenclature). Fragment series c_*n*a_ is isomeric with fragment series c_*n*_ formed via the charge-induced dissociation pathway in Scheme 4.

The remaining, minor MS/MS fragment ions from the linear architecture are accounted for by charge-remote homolytic bond cleavages, yielding radicals that decompose further through typical free radical chemistry involving H^•^ shifts, alkyl rearrangements, and β bond scissions.^8^ Their structures are summarized in Figure 4, and plausible fragmentation mechanisms are presented in Schemes S9 (for series b_*n*_), S10 (for series x_*n*_), and S11 (for series c_*n*b_″). Scheme S9 also includes an alternative, charge-remote pathway to fragments with the same composition as c_*n*_ but having an isomeric connectivity (hence, termed c_*n*a_). The relative intensity of series c_*n*a_ should be similar to those of the other fragments formed through radical intermediates (Figure 3); hence, the PBBM fragments with a total end group mass of 220 Da (marked by purple stars in Figure 3) must mainly originate from the charge-induced dissociation pathway of Scheme 4 and have the dioxanylium terminated structure c_*n*_.

### MS/MS of the Benzoylated Hyperbranched Polymer

**Figure 5:**
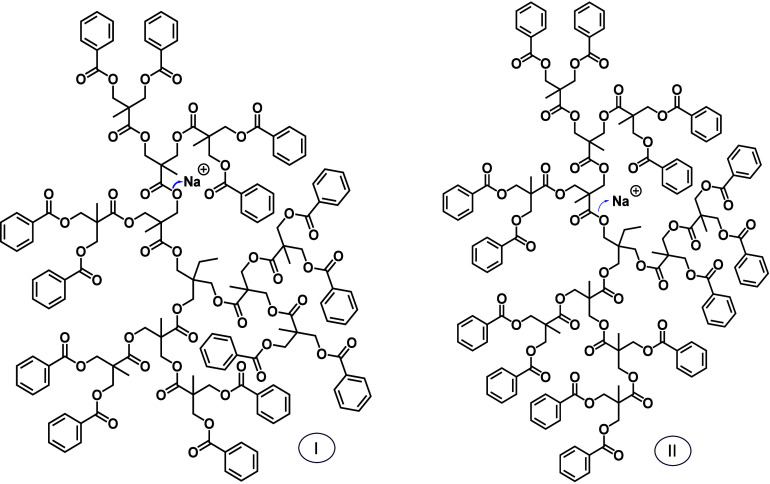
Two isomers (I and II) of a hyperbranched polymer composed of a trimethylolpropane core, 11 bis-MPA units, and 14 benzoyl substituents, viz. TPM-Bis_11_-Bz_14_. Both have the elemental composition C_159_H_158_O_50_ and form [M + Na]^+^ ions at *m*/*z* 2889.8. The blue arrows indicate charge-induced ester bond cleavage to form acylium ions, which are the main MS/MS fragments (cf. Figure 6).

The precursor ion subjected to MS/MS analysis was the fully esterified hyperbranched 11-mer, composed of 11 bis-MPA and 14 benzoyl units. Its [M + Na]^+^ ion is observed at *m*/*z* 2889.8 (Figure S6), which lies within 10% of the *m*/*z* value for the selected dendrimer. In contrast to the dendritic and linear oligomers investigated, which have a single connectivity (primary structure), the hyperbranched oligomer is composed of several different isomers, depending on the type of branching around the trifunctional initiator; two of the possible isomers, termed I and II, are shown in Figure 5 to illustrate this difference.

**Figure 6:**
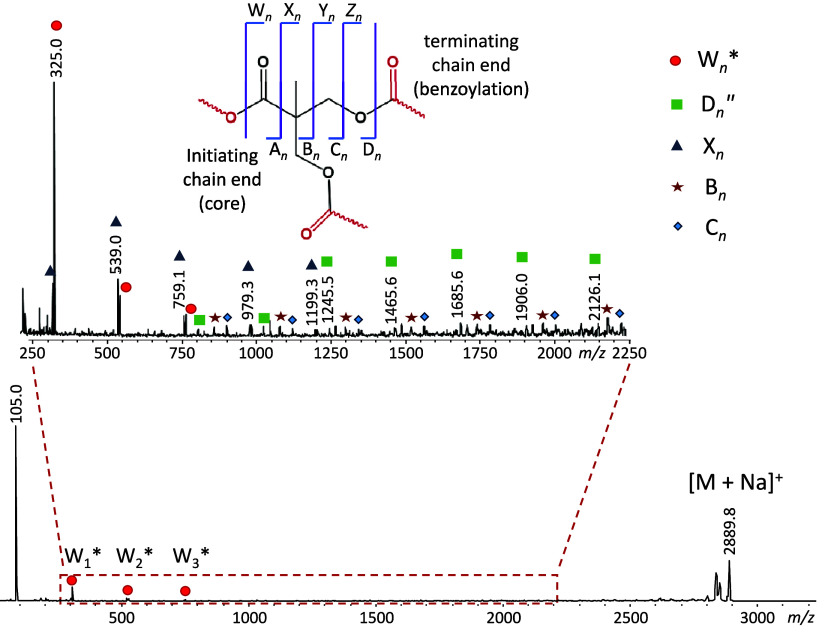
MALDI-ToF MS/MS spectrum of the sodiated, benzoyl-functionalized hyperbranched polymer TMP-Bis_11_-Bz_14_ (*m*/*z* 2889.8). The fragment nomenclature is explained in the top inset; upper-case acronyms are used for branched architectures. The subscripts indicate the number of complete or partial bis-MPA repeat units in the fragment. A double prime indicates fragments with a new saturated chain end, while an asterisk indicates fragment without Na^+^. See schemes for mechanistic rationalizations.

In keeping with both the dendrimer as well as the linear polymer, the dominant MS/MS fragment is the benzoyl cation at *m*/*z* 105.0, cf. Figure 6. Additionally, a series of bis-MPA acylium ions with a 220-Da repeat unit is observed, starting at *m*/*z* 325.0 and extending to *m*/*z* 1205.3 (W_1_*–W_5_*). The acylium ion at *m*/*z* 325.0 (W_1_*) was also formed from the dendrimer, but not the linear polymer. On the other hand, the larger acylium ions (a contiguous W_2_*–W_5_* fragmentation series) constitute a unique MS/MS feature of the hyperbranched polymer.

**Figure 7:**
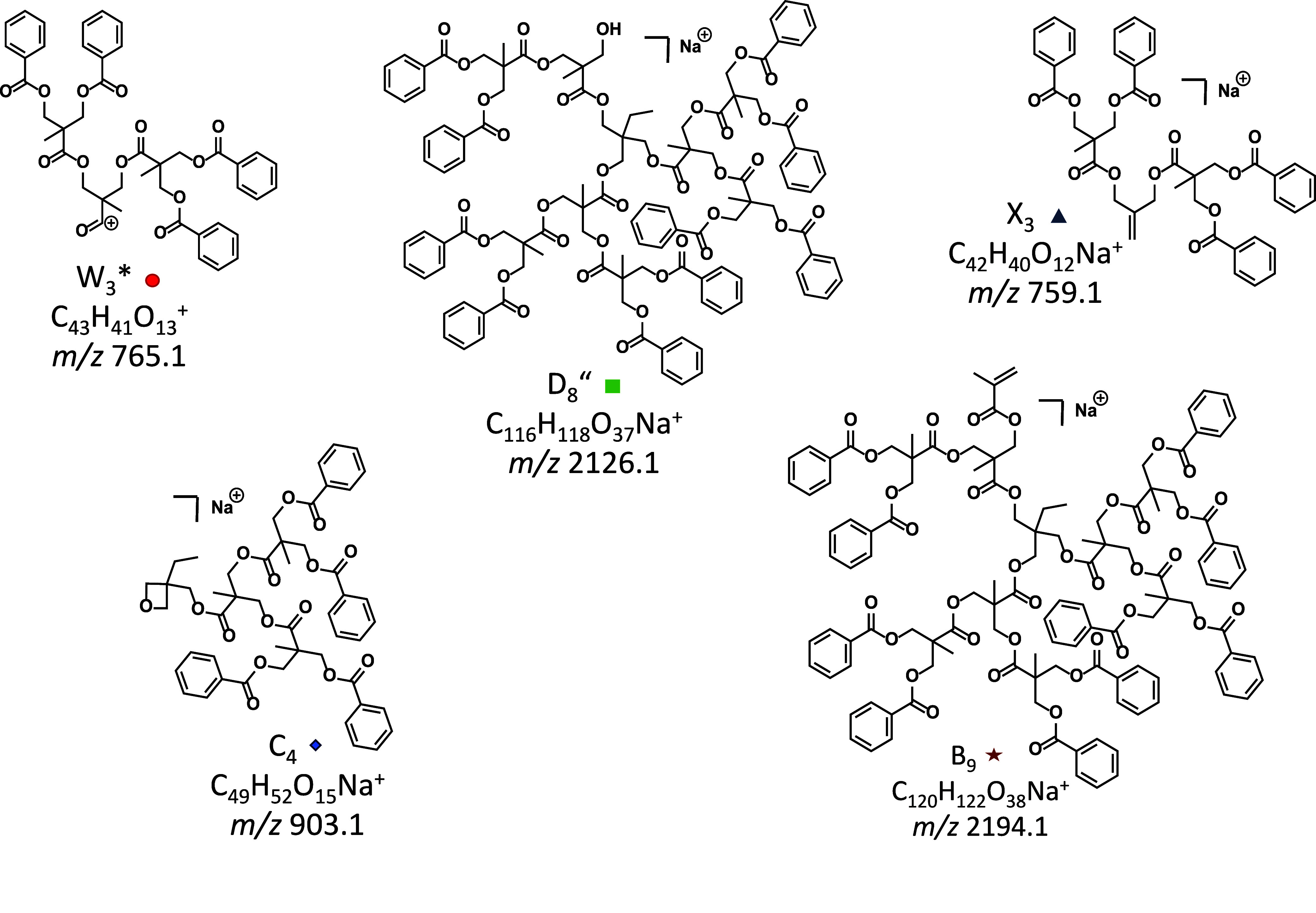
Structures of MS/MS fragments marked in the MALDI-ToF MS/MS spectrum of the sodiated, benzoyl-functionalized hyperbranched polymer TMP-bis_11_-Bz_14_ (*m*/*z* 2889.8), cf. Figure 6. W_n_* (acylium ions), D_*n*_″ (fragments with OH end group), and X_*n*_ series (fragments with alkene end group), represented by W_3_*, D_8_″, and X_3_, formed by charge-induced fragmentation, as shown in Scheme S12; C_*n*_ series (fragments with oxetane end group), represented by C_4_ formed by charge-induced fragmentation according to Scheme S13; B_*n*_ series (fragments with isopropenyl end group), represented by B_9_ formed by charge-remote homolytic cleavages according to the mechanism in Scheme S7. The subscripts give the number of complete or partial bis-MPA units.

The W_n_* fragments can be accounted for by charge-induced heterolytic ester bond cleavages in the bis-MPA units, promoted by the Na^+^ Lewis acid, as shown by the blue arrows in Figure 5. Following the mechanisms presented in Schemes 2 and S3, such bond cleavages yield acylium cations (W_n_* series), by loss of the complementary hyperbranched piece as a sodium alkoxylate, or hyperbranched sodiated fragments with alcohol end groups after proton transfer from the acylium cation to the sodium alkoxylate (D_*n*_″ series; green squares). Alternatively, the acylium cation may undergo CO loss and proton/sodium exchange with the departing sodium alkoxylate to form an X_*n*_ fragment with a terminal alkene group (cf. Scheme S12 and Figure 7); this fragment series is marked with blue triangles in Figure 6.

Additionally, two minor fragment series were identified, viz. B_*n*_ and C_*n*_, which are marked with red stars and blue diamonds, respectively (see proposed structures in Figure 7). The C_*n*_ ions are attributed to charge-induced rearrangement at the core, leading to the expulsion of CO plus a carboxylic acid moiety to generate a truncated hyperbranched fragment with an oxetane substituent. This process is demonstrated in Scheme S13 for the formation of C_4_; oxetane ring formation has been observed in the MS/MS fragmentation of alkali metalated ceramides.^37^ Conversely, the B_*n*_ ions are attributed to charge-remote radical losses that generate fragments with isopropenyl end groups (cf. Figure 7), similar to the fragmentation pathway observed for the dendrimer, which is shown in Scheme S7.

## Conclusions

The MS/MS spectra of the three architectures investigated exhibit both common and distinct features. For all three architectures, the benzoyl cation (*m*/*z* 105) is the most abundant fragment. Although all three architectures have the same nominal repeat unit of 220 Da, only the linear polymer contains a benzoyl substituent at every bis-MPA moiety. This unique feature results in COO–CH_2_ bond scission at each repeat unit, leading to a contiguous fragment series over the entire mass range of the selected *n*-mer (Figure 3). In sharp contrast, the dendrimer does not produce any contiguous fragment series but instead selectively fragments at the core, interior, and exterior sites. This reactivity is ascribed to the propensity to relieve crowding and steric hindrance within dendrimer species.

The hyperbranched polymer more closely resembles the dendrimer in chain connectivity than the linear polymer (vide supra) but still forms homologous fragment series in the MS/MS experiments similar to the linear polymer. This is most likely the result of having a mixture of different hyperbranched isomers instead of one single structure, as with the dendrimer. These isomers have different branching features, each favoring its own selective cleavages, resulting in fragment series from all possible isomers. Thus, while only one bis-MPA acylium ion is observed from the dendrimer, the hyperbranched polymer generates a distribution of such fragments with up to five bis-MPA units. Meanwhile, the linear polymer only shows the benzoyl cation and no other acylium species. Hence, the number of acylium structured fragments enables differentiation among the three different architectures.

## Supporting Information Available

Synthetic schemes of the polymers studied; mechanistic rationalizations of the MS/MS fragmentation channels of dendritic, linear, and hyperbranched ions; MS/MS fragment structures; zoom-in views of MS/MS spectra; MALDI-ToF MS, GPC, and NMR spectra of the polymers (PDF)
