# Supplementary material for: Tandem Mass Spectrometry Reflects Architectural Differences in Analogous, Bis-MPA-Based Linear Polymers, Hyperbranched Polymers, and Dendrimers
Source: J Am Soc Mass Spectrom. 2024 Nov 8;35(12):3135–46. doi: 10.1021/jasms.4c00330 (PMC11622245; doi:10.1021/jasms.4c00330)
Supplement: Supplementary file 1 — js4c00330_si_001.pdf [file js4c00330_si_001.pdf]

Supporting Information for:

Tandem Mass Spectrometry Reflects Architectural Differences in Analogous, Bis-MPA-based Linear Polymers, Hyperbranched Polymers, and Dendrimers

Kayla Williams-Pavlantos[1,#] | McKenna J. Redding[2,#] (ORCID: 0000-0002-2656-8978) | Oluwapelumi O. Kareem[2] | Mark A. Arnould[3] | Scott M. Grayson[2,*] (ORCID: 0000-0001-6345-8762) | Chrys Wesdemiotis[1,*] (ORCID: 0000-0002-7916-4782)

[1]Department of Chemistry, University of Akron, Akron, Ohio 44325, United States

[2]Department of Chemistry, Percival Stern Hall, Tulane University, New Orleans, Louisiana 70118, United States

[3]Bruker Scientific LLC., 40 Manning Road, Billerica, MA 01821, United States

[#] These authors contributed equally to this project.

Correspondence

S. M. Grayson, Department of Chemistry, Percival Stern Hall, Tulane University, New Orleans, Louisiana 70118, United States.

Email: sgrayson@tulane.edu

C. Wesdemiotis, Department of Chemistry, University of Akron, Akron Ohio 44325, United States

Email: wesdemi@uakron.edu

Table of contents:

Scheme S1. Synthetic scheme of Tetra[G2]Bz_{16}.

Scheme S2. Synthetic scheme for benzoyl functionalized hyperbranched polymer.

Scheme S3. Mechanistic rationalization for the formation of fragment D_I'' from sodiated Tetra[G2]Bz_{16}

Scheme S4. Mechanistic rationalization for the loss of $C_7H_4O_2$ (120 Da) from sodiated Tetra[G2]Bz_{16}.

Scheme S5. Mechanistic rationalization for the loss of $C_8H_6O_2$ (134 Da) from sodiated Tetra[G2]Bz_{16}.

Scheme S6. Mechanistic rationalization for the consecutive loss of $C_7H_4O_2$ (120 Da) from the fragment formed after the loss of $C_8H_6O_2$ (134 Da) from sodiated Tetra[G2]Bz_{16} (overall loss of 134 + 120 = 254 Da).

Scheme S7. Mechanistic rationalization for the formation of fragment B_I from sodiated Tetra[G2]Bz_{16}.

Scheme S8. Mechanistic rationalization for the formation of fragment B_C from sodiated Tetra[G2]Bz_{16}.

Scheme S9. Mechanistic rationalization for the formation of fragment series b_n (EGs 86 Da) from sodiated PBBM.

Scheme S10. Mechanistic rationalization for the formation of fragment series x_n (EGs 134 Da) from sodiated PBBM.

Scheme S11. Mechanistic rationalization for the formation of fragment series c_{nb}'' (EGs 102 Da) from sodiated PBBM.

Scheme S12. Mechanistic rationalization for the formation of fragment series W_n*, D_n'', and X_n from sodiated TMP-Bis_{11}-Bz_{14}.

Scheme S13. Mechanistic rationalization for the formation of fragment series C_n from sodiated TMP-Bis_{11}-Bz_{14}.

Figure S1: MALDI-ToF MS and GPC analysis of Tetra[G2]Bz_{16}.

Figure S2: ^{1}H NMR spectrum of Tetra[G2]Bz_{16}.

Figure S3. MALDI-ToF MS and GPC analysis of PBBM linear polymer.

Figure S4. ^{1}H NMR spectrum of PBBM linear polymer.

Figure S5. ^{1}H NMR spectrum of the deprotected hyperbranched polymer, the precursor for the benzoyl functionalized hyperbranched polymer.

Figure S6. MALDI-ToF MS and GPC analysis of TMP-Bis_n-Bz_{n+3} hyperbranched polymer.

Figure S7. ^{1}H NMR spectrum of TMP-Bis_n-Bz_{n+3} hyperbranched polymer.

Figure S8. Fragments D_C'' and D_E'' in the MS/MS spectrum of sodiated Tetra[G2]Bz_{16}.

Figure S9. Low mass region of the MALDI-ToF MS/MS spectrum of sodiated PBBM 14-mer.

Figure S10. High mass region of the MALDI-ToF MS/MS spectrum of sodiated PBBM 14-mer.

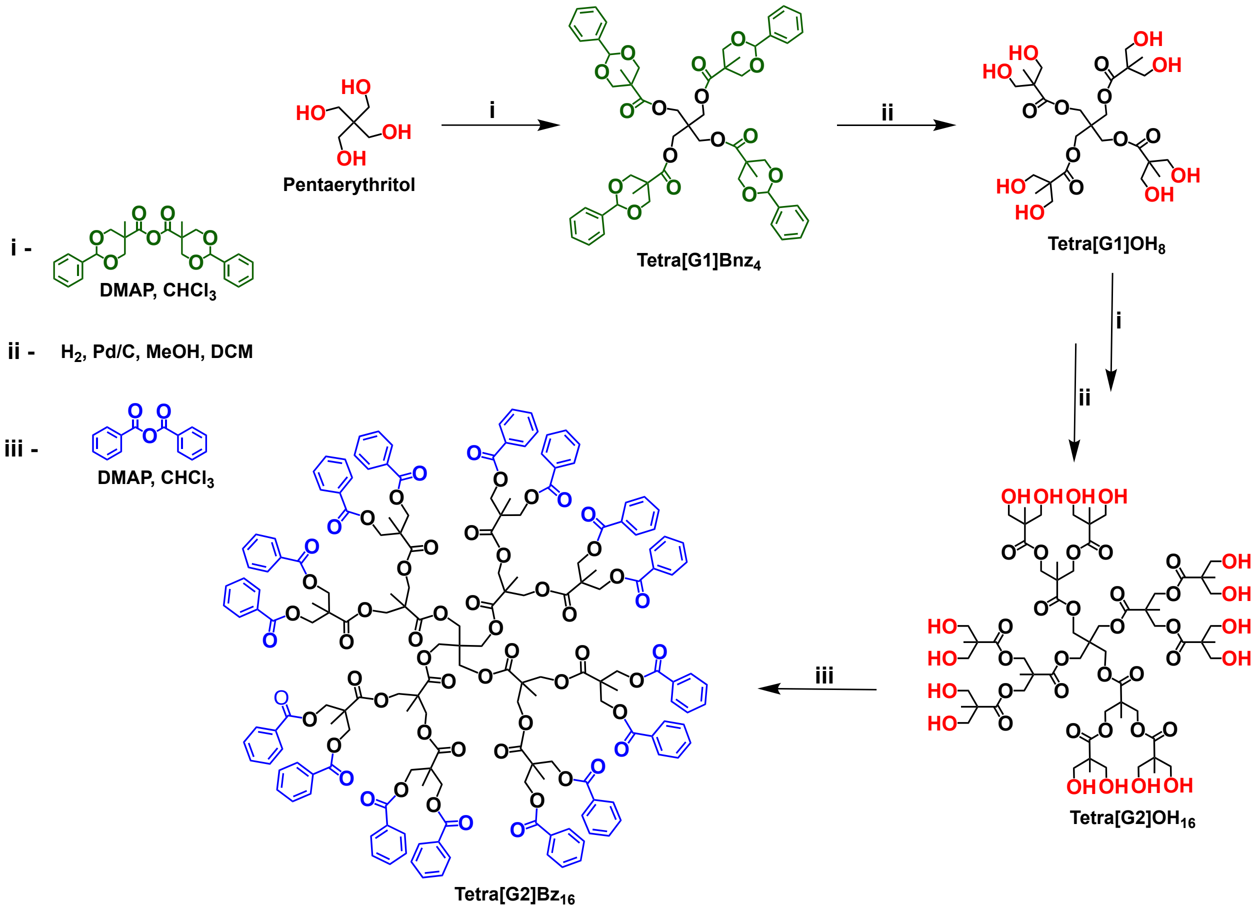

Scheme S1. Synthetic scheme for Tetra[G2]Bz_{16}.

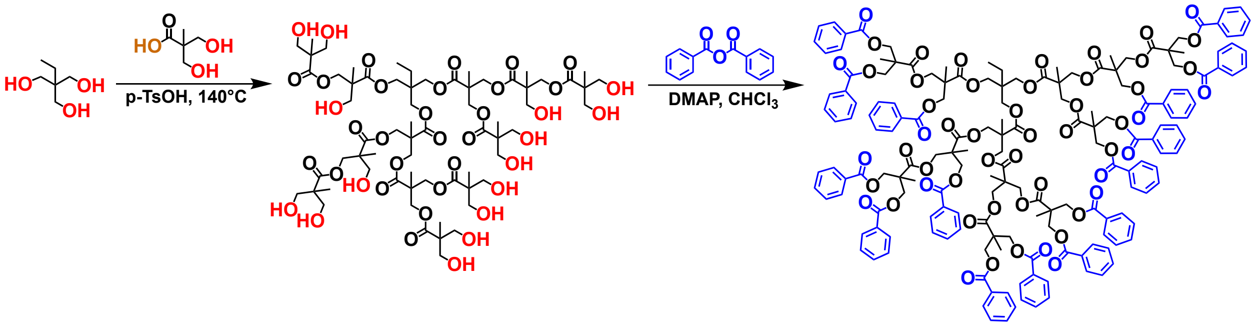

Scheme S2. Synthetic scheme for benzoyl functionalized hyperbranched polymer.

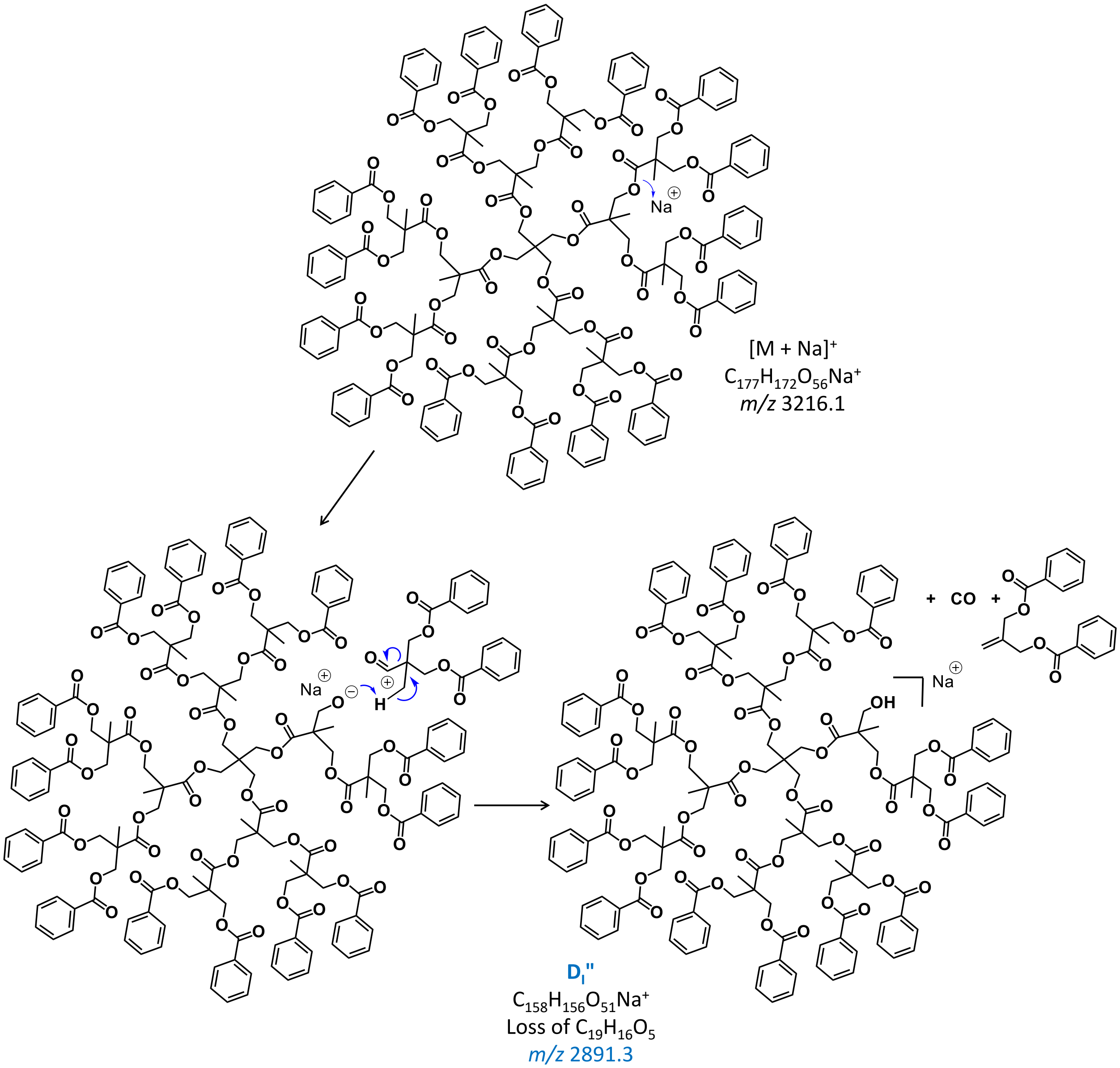

Scheme S3. Charge-induced bond cleavage of an interior ester bond in the sodiated dendrimer (top), leading to an acylium ion that interacts electrostatically with a sodium alkoxylate (bottom, left), followed by proton abstraction from the acylium ion by the alkoxylate and expulsion of CO + 2-methylene-1,3-propylene dibenzoate (overall loss of $C_{19}H_{16}O_5$, 324.1 Da), to form a truncated dendrimer fragment D_I" with a primary OH group at an interior site (bottom, right).

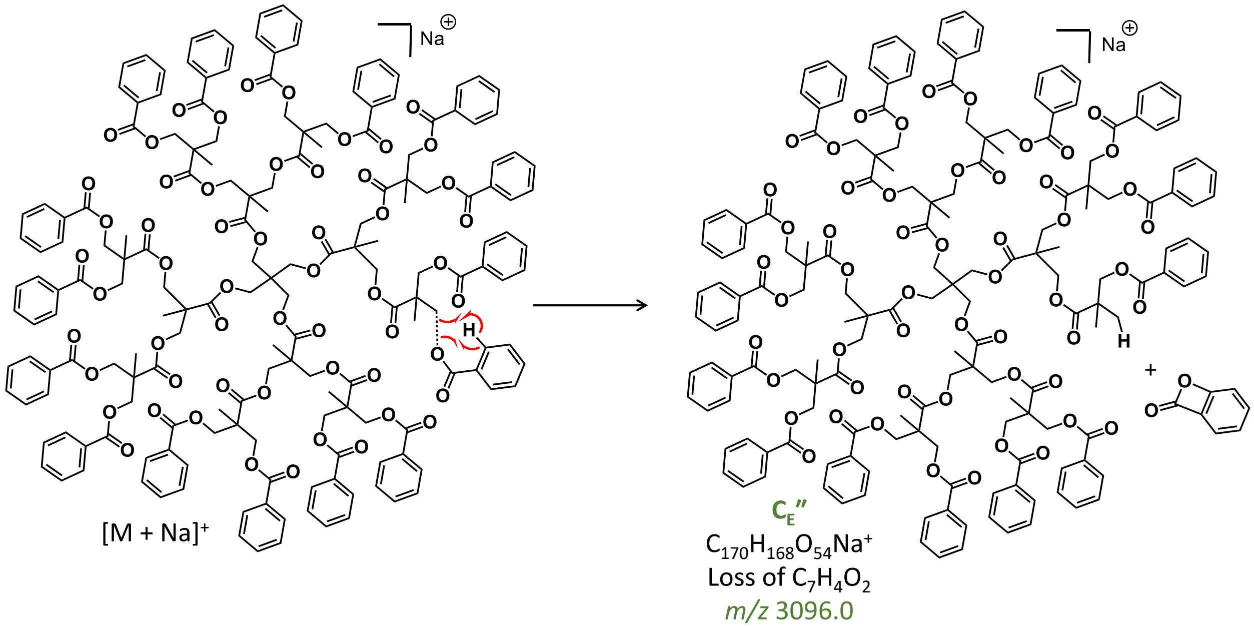

Scheme S4. Charge-remote homolytic bond cleavage accompanied by H-rearrangement in the periphery of the sodiated Tetra[G2]Bz_{16} dendrimer, resulting in the loss of $C_7H_4O_2$ (120.0 Da) and the fragment C_E'' at *m/z* 3096.0.

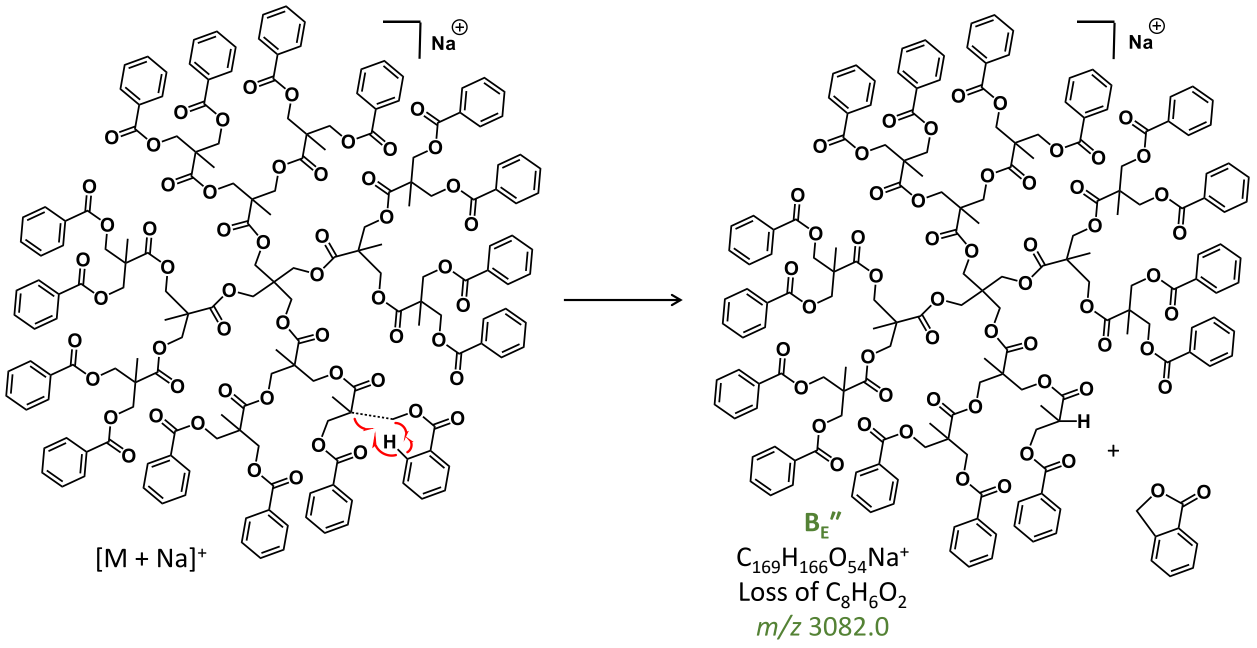

Scheme S5. Charge-remote homolytic bond cleavage accompanied by H-rearrangement in the periphery of the sodiated Tetra[G2]Bz_{16} dendrimer, resulting in the loss of $C_8H_6O_2$ (134.0 Da) and the fragment B_E'' at *m/z* 3082.0.

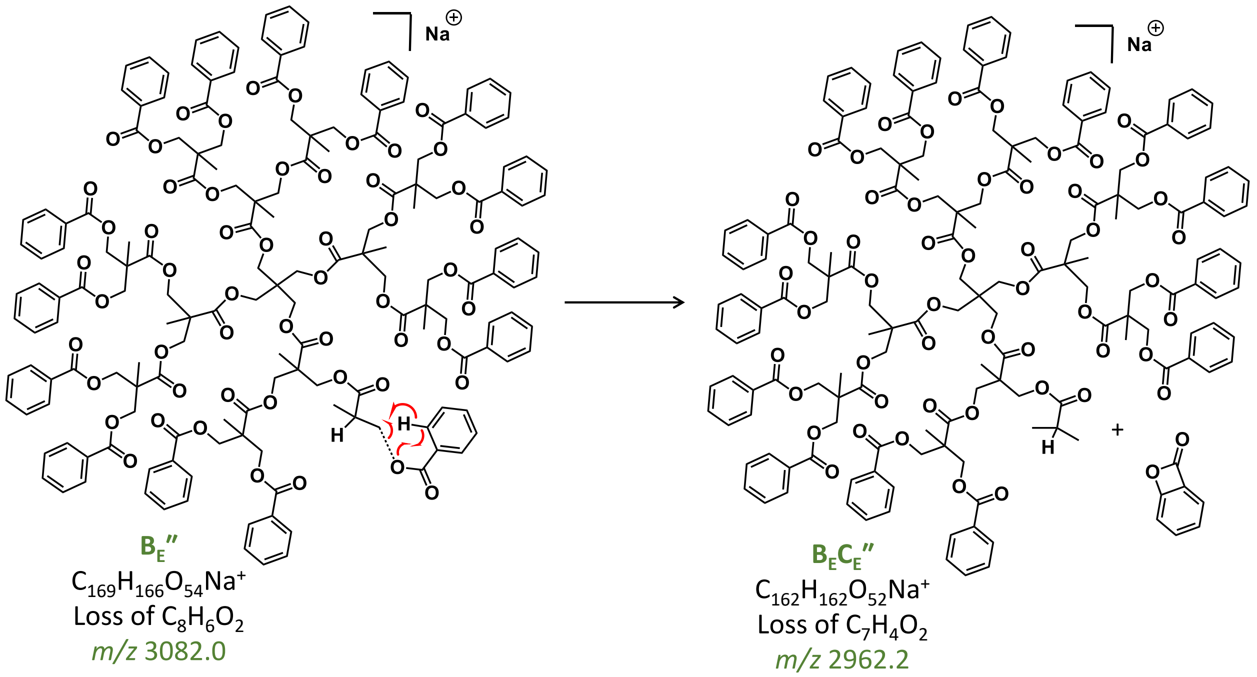

Scheme S6. Consecutive charge-remote homolytic bond cleavage accompanied by H-rearrangement in the periphery of dendritic fragment B_E'' (Scheme S5), resulting in the loss of $C_7H_4O_2$ (120.0 Da) and the fragment B_EC_E'' at *m/z* 2962.2; the same fragment can be formed by consecutive $C_8H_6O_2$ (134.0 Da) loss from C_E'' (Scheme S4). The consecutive dissociations leading to B_EC_E'' (*m/z* 2962.2) can take place at the mono-benzoylated bis-MPA unit of B_E'' (or C_E''), as shown in this Scheme, or at any other benzoyl pendant. The much higher relative abundance of B_EC_E'' compared to either B_E'' or C_E'' (cf. Figure 1) strongly suggests that consecutive fragmentation at the same bis-MPA to yield a saturated terminal ester unit with an isopropyl substituent is favored.

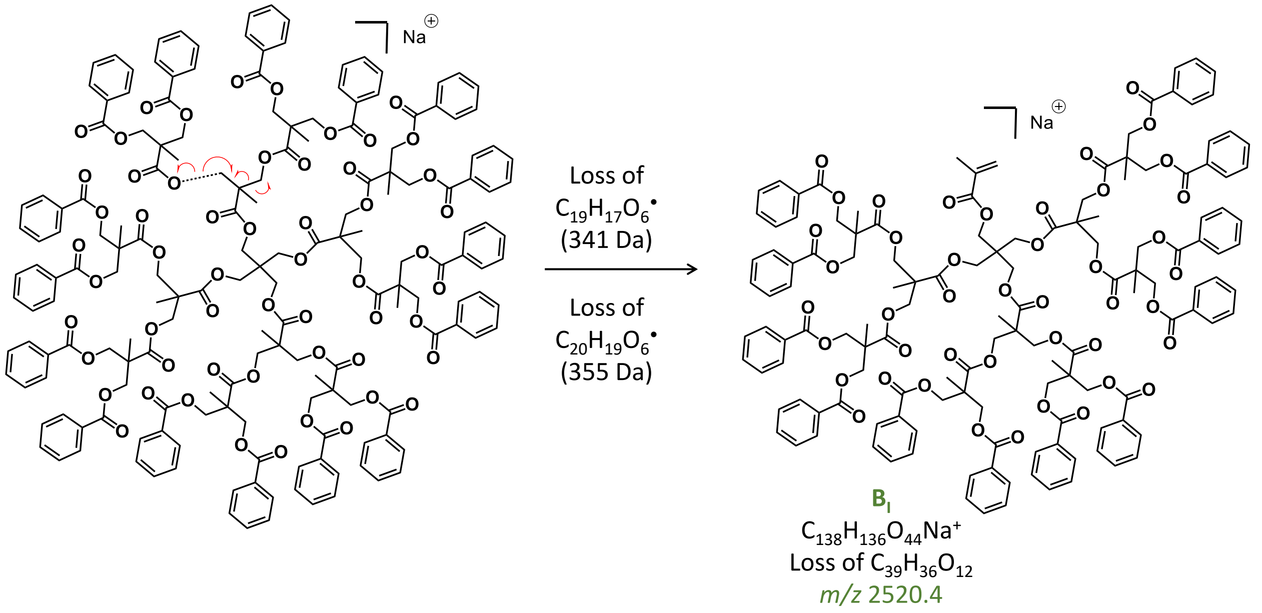

Scheme S7. Charge-remote homolytic C-O bond cleavage at an interior bis-MPA unit of the sodiated Tetra[G2]Bz_{16} dendrimer, releasing a $C_{19}H_{17}O_6^{\bullet}$ (341.1 Da) branch, and consecutive β C-C bond scission in the emerging dendritic intermediate, releasing an additional $C_{20}H_{19}O_6^{\bullet}$ branch (355.1 Da) to form fragment B_I at *m/z* 2520.4 (overall loss of $C_{39}H_{36}O_{12}$, 696.2 Da).

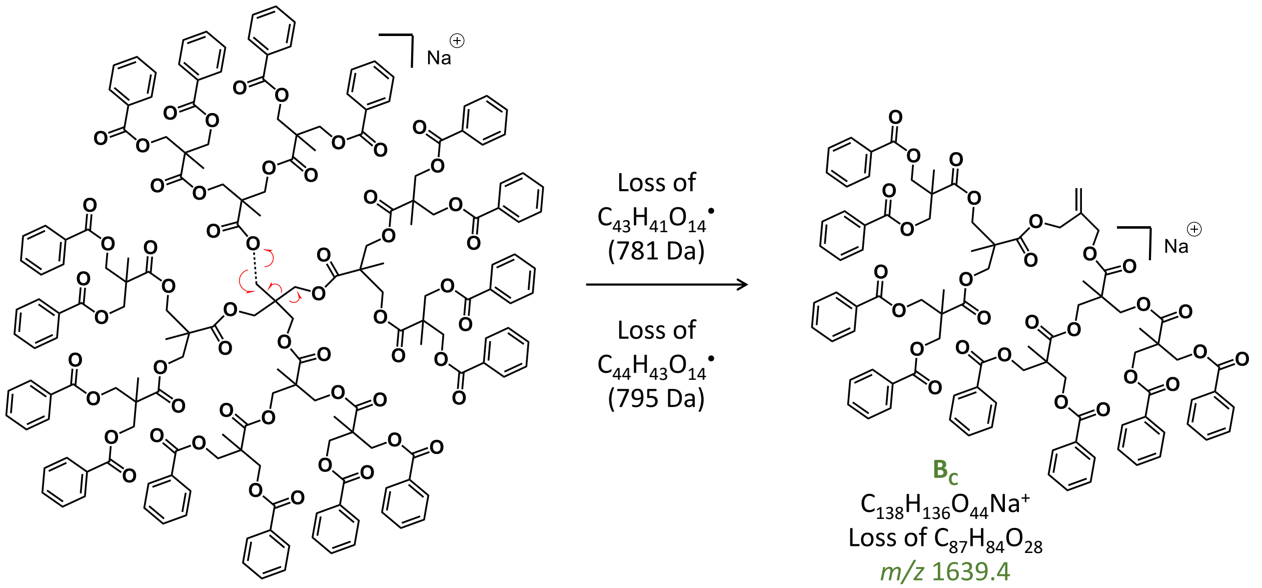

Scheme S8. Charge-remote homolytic C-O bond cleavage at the core of the sodiated Tetra[G2]Bz_{16} dendrimer, releasing a $C_{43}H_{41}O_{14}^{\bullet}$ (781.2 Da) branch, and consecutive β C-C bond scission in the emerging dendritic intermediate, releasing an additional $C_{44}H_{43}O_{14}^{\bullet}$ branch (795.3 Da) to form fragment B_C at *m/z* 1639.4 (overall loss of $C_{87}H_{84}O_{28}$, 1576.5 Da).

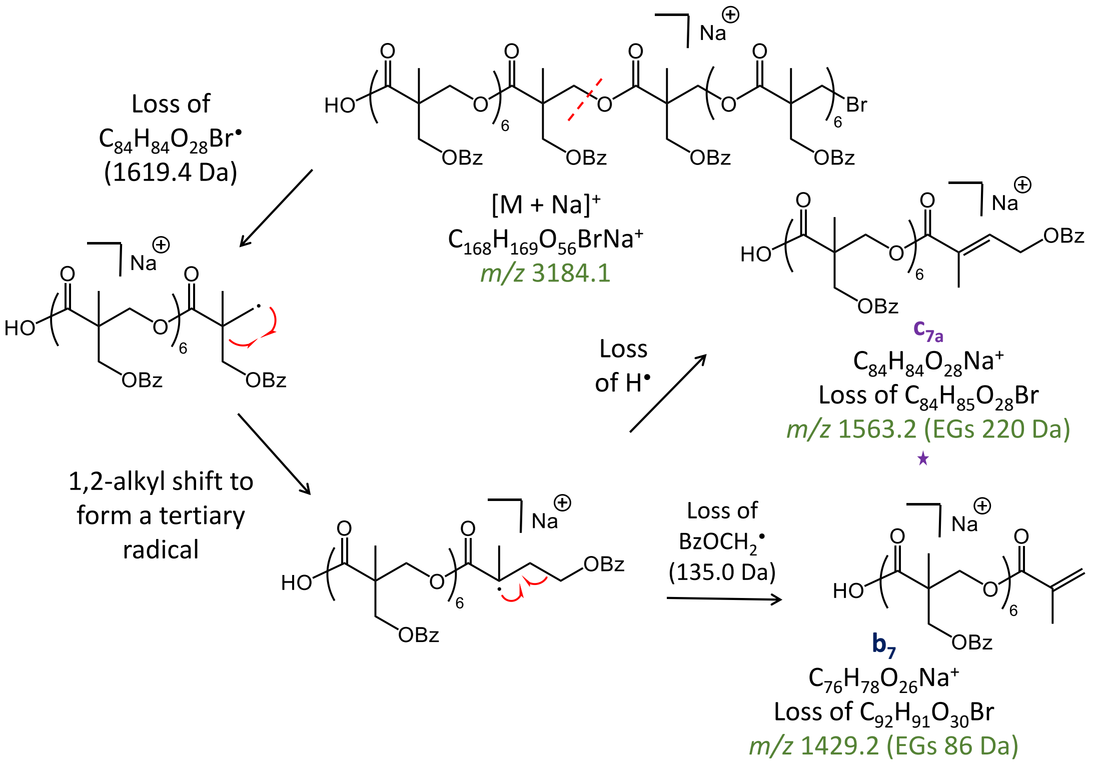

Scheme S9. Charge-remote homolytic bond cleavage at the 7th CH_2-OCO bond of the sodiated PBBM 14-mer, expelling a carboxy terminated radical to form an incipient fragment ion with a primary alkyl radical site, 1,2-alkyl shift to form a more stable tertiary (and resonance-stabilized) radical site, and consecutive β C-H or β C-C bond scission leading to fragment ions c_{7a} and b_7, respectively. A similar process at the other CH_2-OCO bonds gives rise to the fragment ion distributions c_{na} and b_n (cf. Figure 3). Note that fragment series c_{na} is isomeric with c_n formed by the charge-induced dissociation pathway depicted in Scheme 4; the small relative intensity of b_n (marked with dark blue triangles in Figure 3) and of all other fragments generated via charge-remote homolytic bond cleavages (Schemes S10-S11) strongly suggests that c_{na} constitutes only a small fraction of the observed c_n series (marked with purple stars in Figure 3).

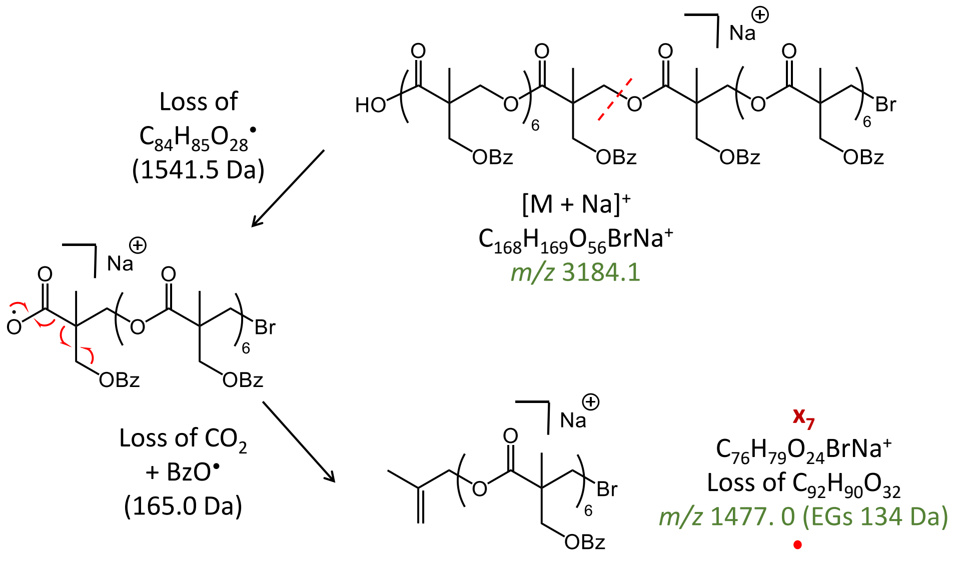

Scheme S10. Charge-remote homolytic bond cleavage at the 7th CH_2-OCO bond of the sodiated PBBM 14-mer, expelling an alkyl terminated radical to form an incipient fragment ion with a carboxy radical chain end and consecutive β C-C and C-O bond scissions releasing CO_2 and a $BzO^{\bullet}$ radical, respectively, to generate fragment x_7. A similar process at the other CH_2-OCO bonds gives rise to the fragment ion distribution x_n, marked with red circles in Figure 3.

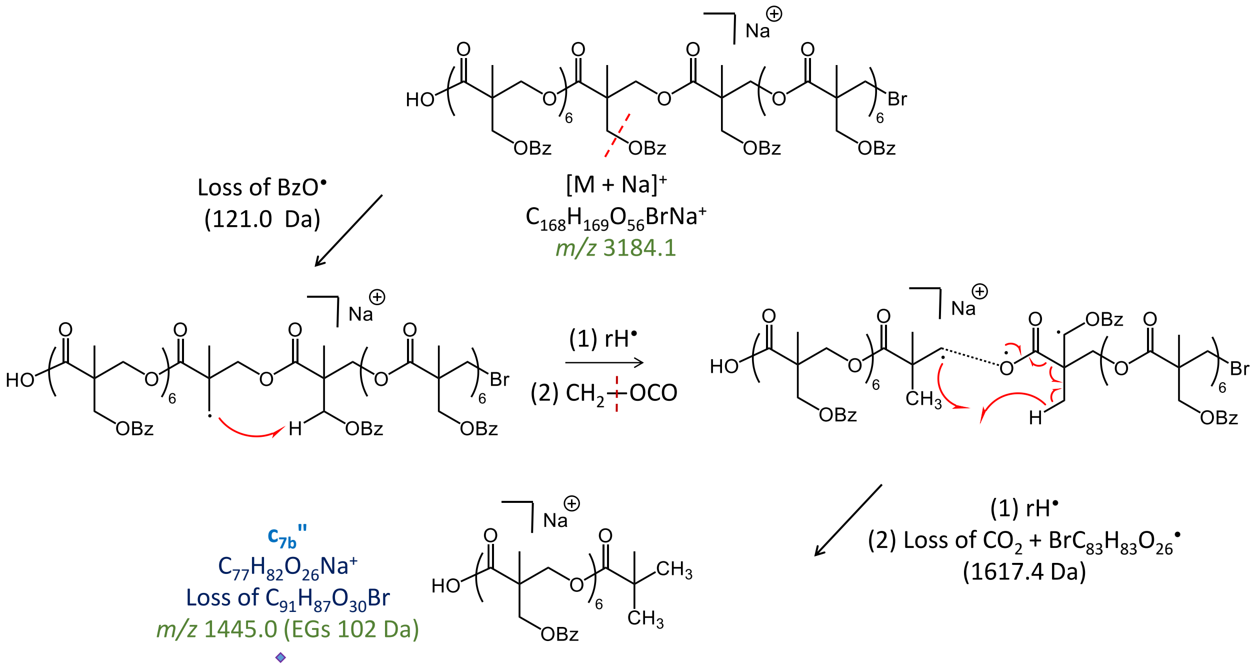

Scheme S11. Charge-remote homolytic bond cleavage in the side chain of the 7th repeat unit of sodiated PBBM 14-mer, expelling a $BzO^{\bullet}$ radical to form an incipient fragment ion with a primary alkyl radical site, intramolecular H-shift moving the unpaired electron next to the benzoyl group, and consecutive H-rearrangement fragmentation via a 6-membered ring that detaches CO_2 and the allylic radical $C_{83}H_{82}O_{26}Br^{\bullet}$ (1573.4 Da) to generate fragment c_{7b}''. A similar process initiated by $BzO^{\bullet}$ loss at the other repeat units gives rise to the fragment ion distribution c_{nb}'', marked by blue diamonds in Figure 3.

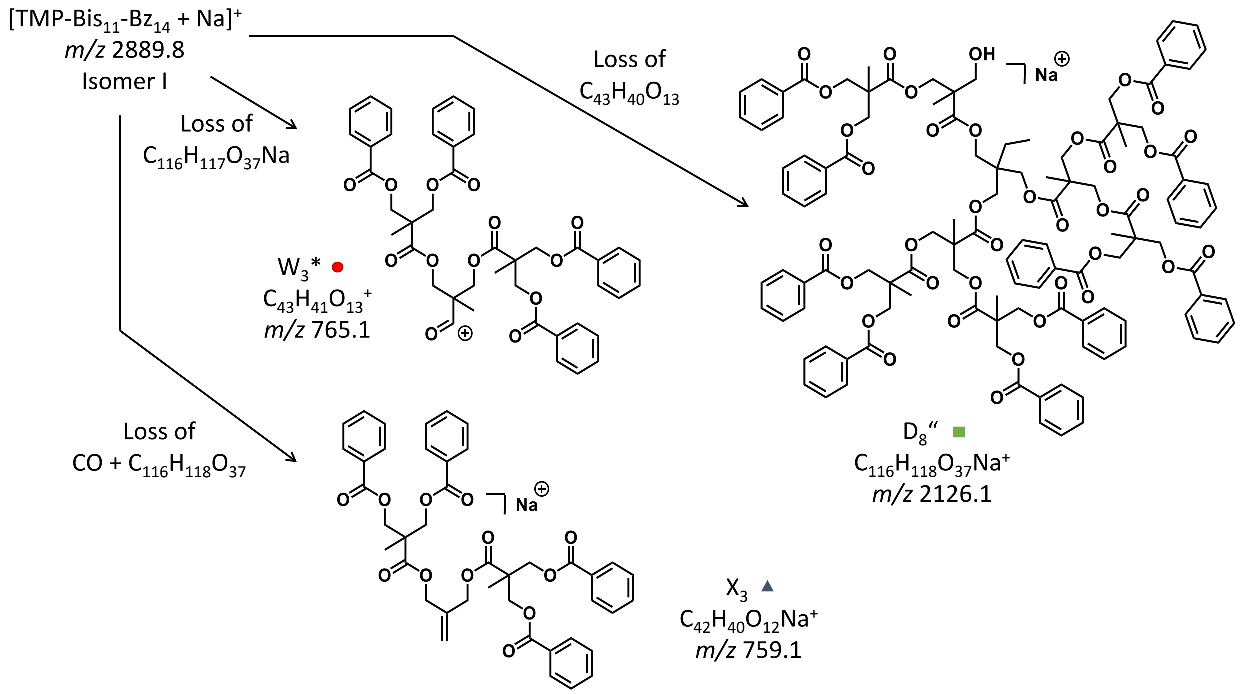

Scheme S12. Charge-induced fragmentation in the sodiated hyperbranched polymer (cf. Figure 6), leading to acylium cations (series W$_n$*) by elimination of a sodium alkoxylate neutral fragment or, after proton transfer from the acylium cation to the carboxylate salt, to truncated sodiated hyperbranched fragments with hydroxy end groups (series D$_n$"). CO elimination from the acylium cation and H^+/Na^+ exchange with the departing carboxylate salt can alternatively generate truncated sodiated hyperbranched fragments with alkene end groups (series X$_n$)

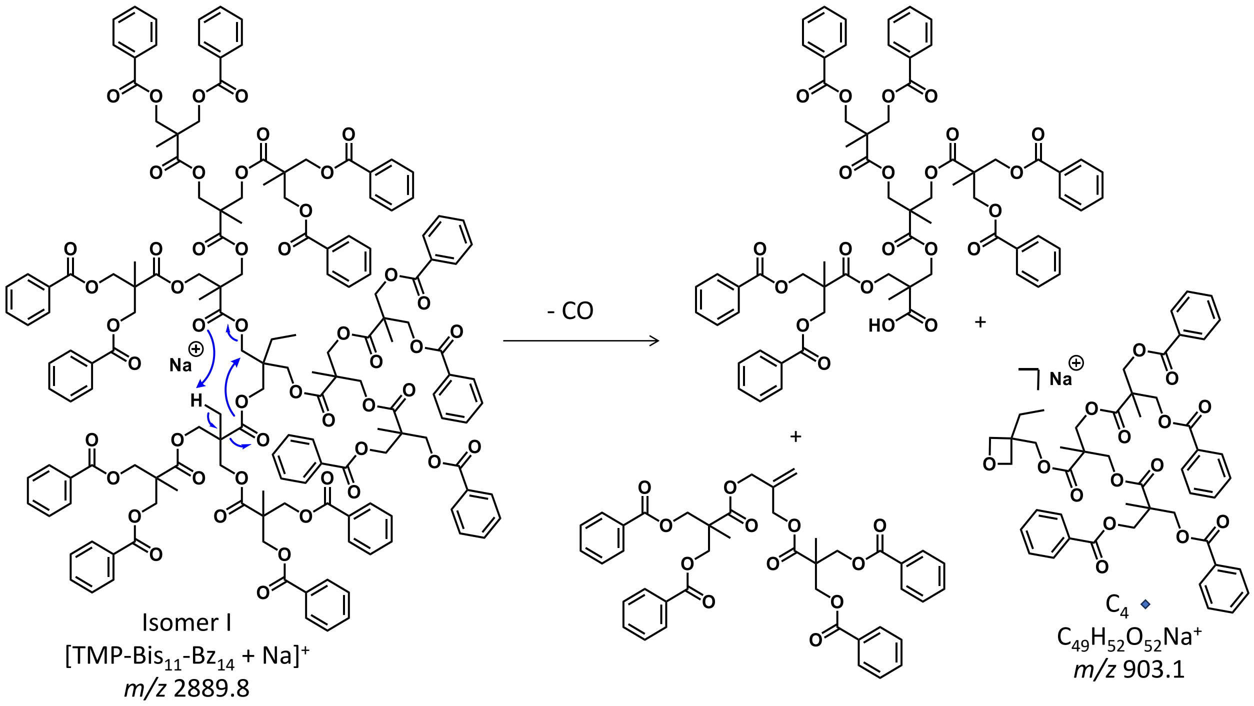

Scheme S13. Charge-induced rearrangement elimination at the core of the sodiated hyperbranched polymer, leading to the expulsion of carboxylic acid and alkene terminated molecules plus CO to yield a truncated hyperbranched fragment C$_4$ with a new oxetane end group. Similar fragmentations at the other core sites and other isomers give rise to series C$_n$ marked with blue diamonds in the MS/MS spectrum of Figure 6.

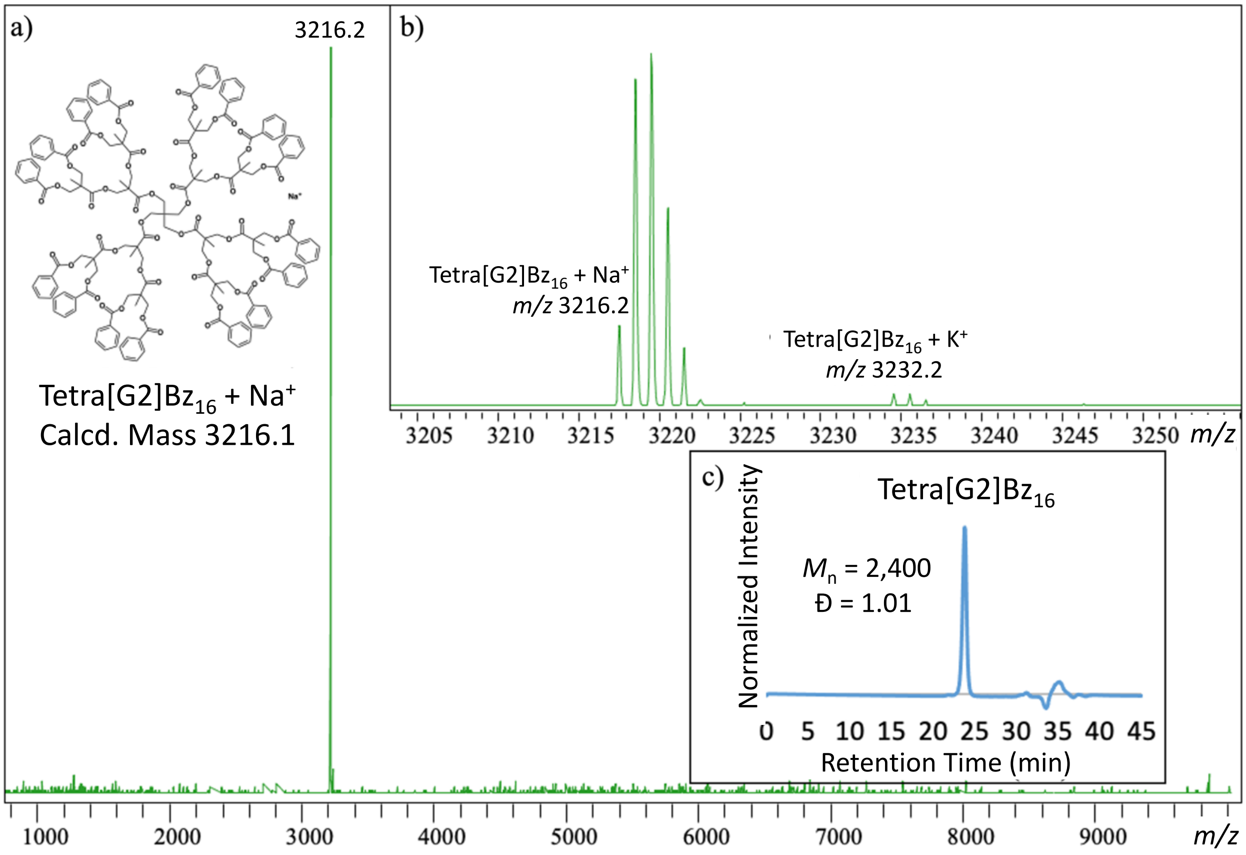

Figure S1. a) MALDI-ToF MS spectrum of Tetra[G2]Bz_{16} with structure, b) zoom view of the *m/z* 3200-3300 region of the MS spectrum, and c) GPC analysis of Tetra[G2]Bz_{16} (M_n = 2,400; Đ = 1.01). All masses or *m/z* ratios marked correspond to monoisotopic values.

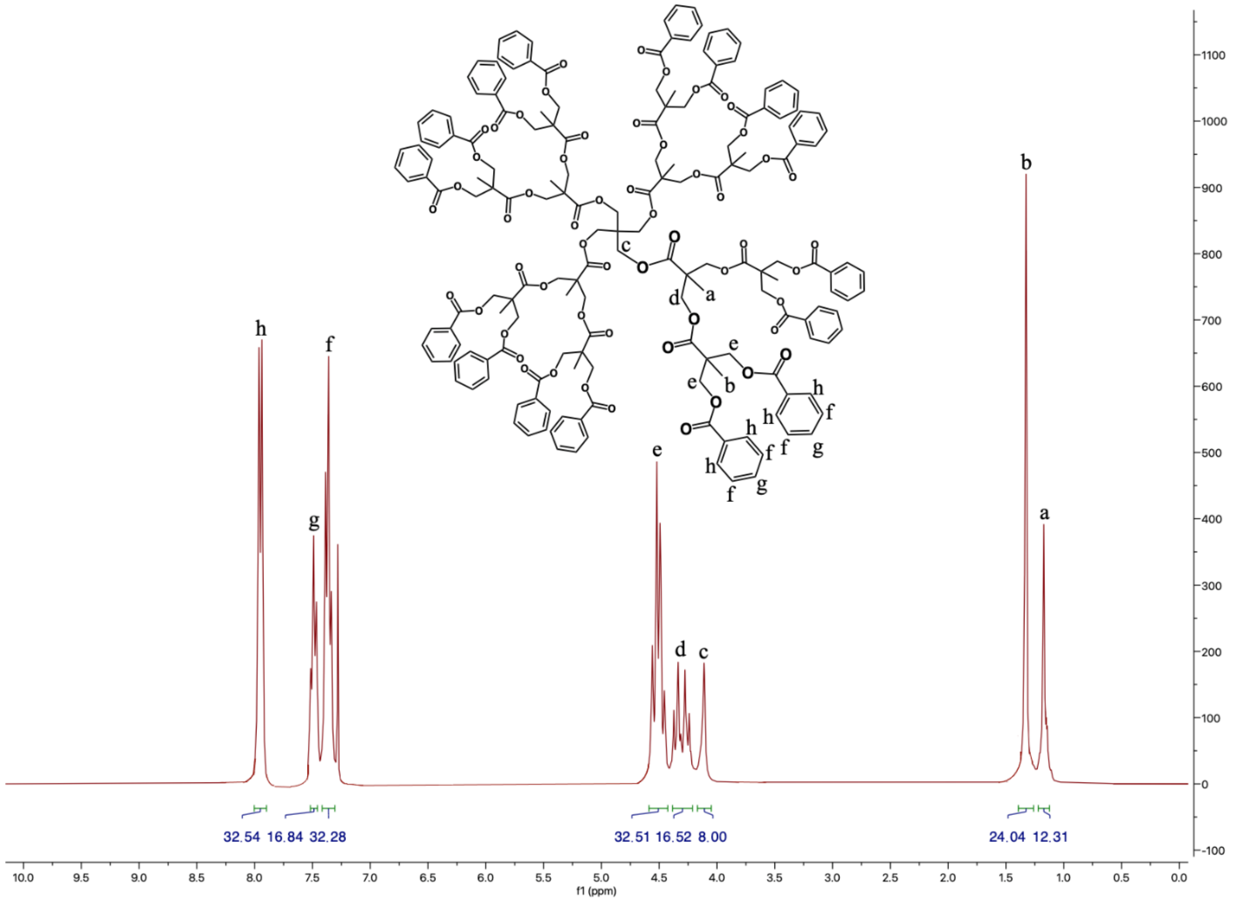

Figure S2. ^{1}H NMR spectrum of Tetra[G2]Bz_{16} with peaks observed labeled on the structure.

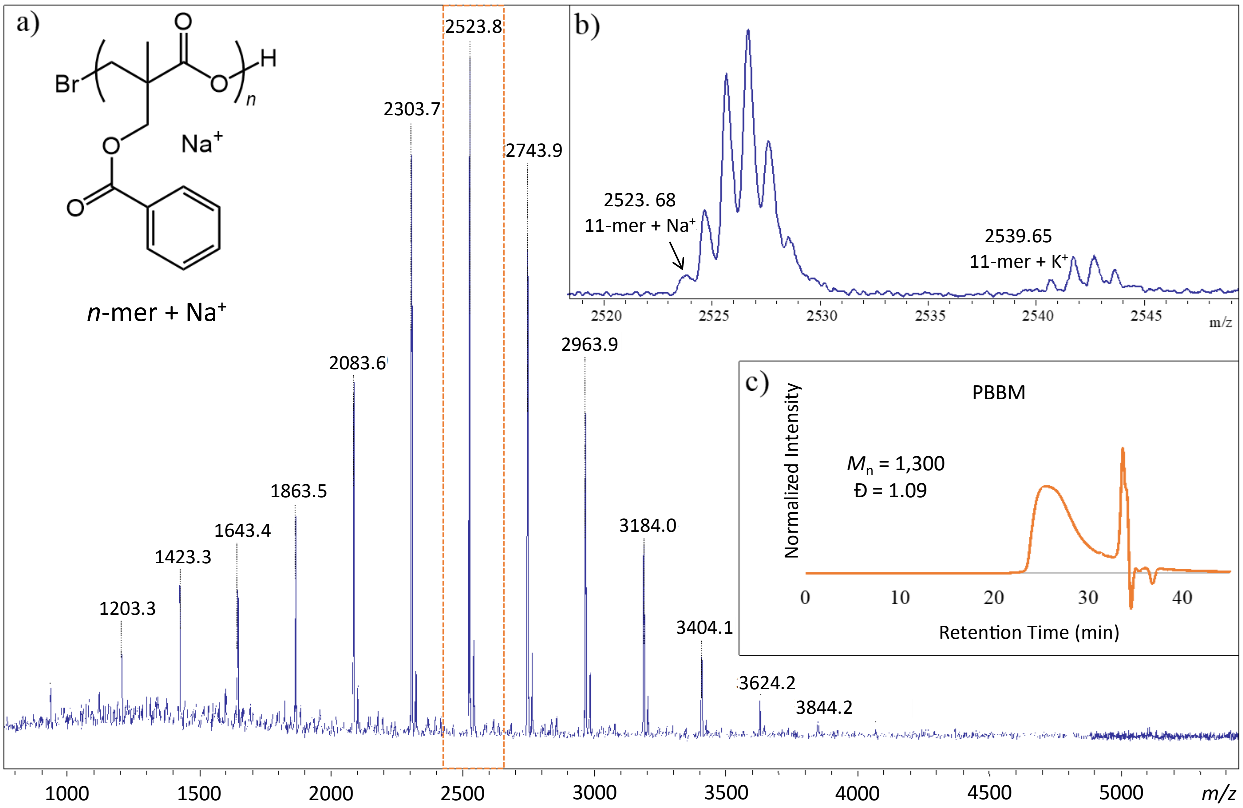

Figure S3. a) MALDI-ToF MS spectrum of poly(3-(benzoyloxy)-2-(bromomethyl)-2-methylpropanoic acid) (PBBM) linear polymer with structure, b) zoomed view of the *m/z* region showing the $[PBBM_{11} + Na]^+$ and $[PBBM_{11} + K]^+$ ions, and c) GPC analysis of PBBM (M_n = 1,300; Đ = 1.09). All *m/z* ratios marked correspond to monoisotopic values.

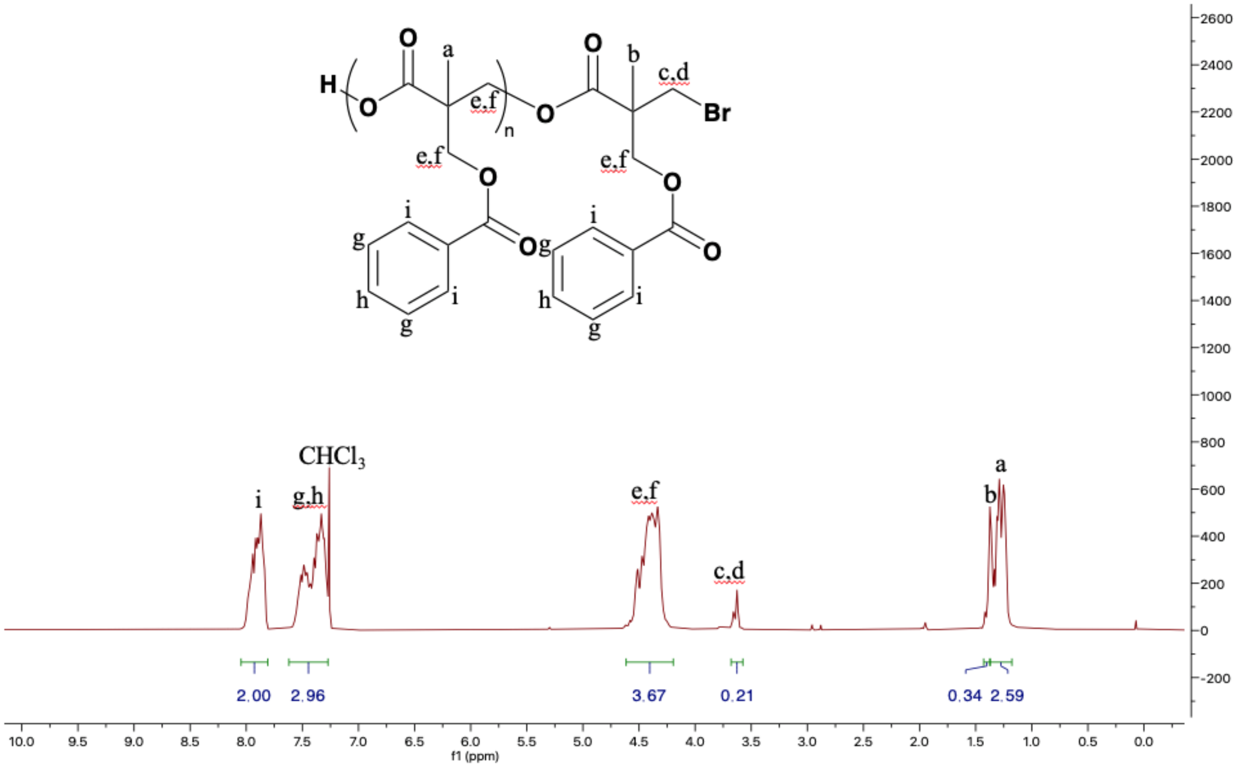

Figure S4. ^{1}H NMR spectrum of PBBM linear polymer with peaks observed labeled in the structure.

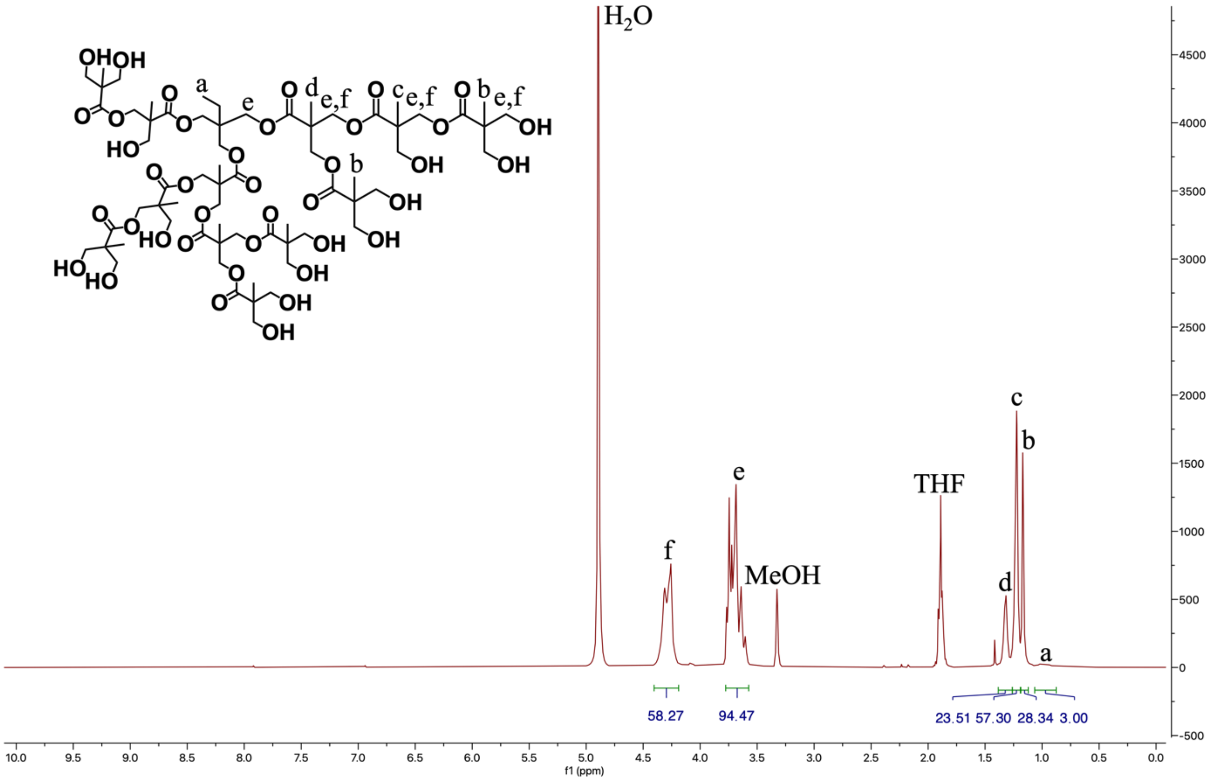

Figure S5. ^{1}H NMR spectrum of the deprotected hyperbranched polymer, the precursor for the benzoyl functionalized hyperbranched polymer.

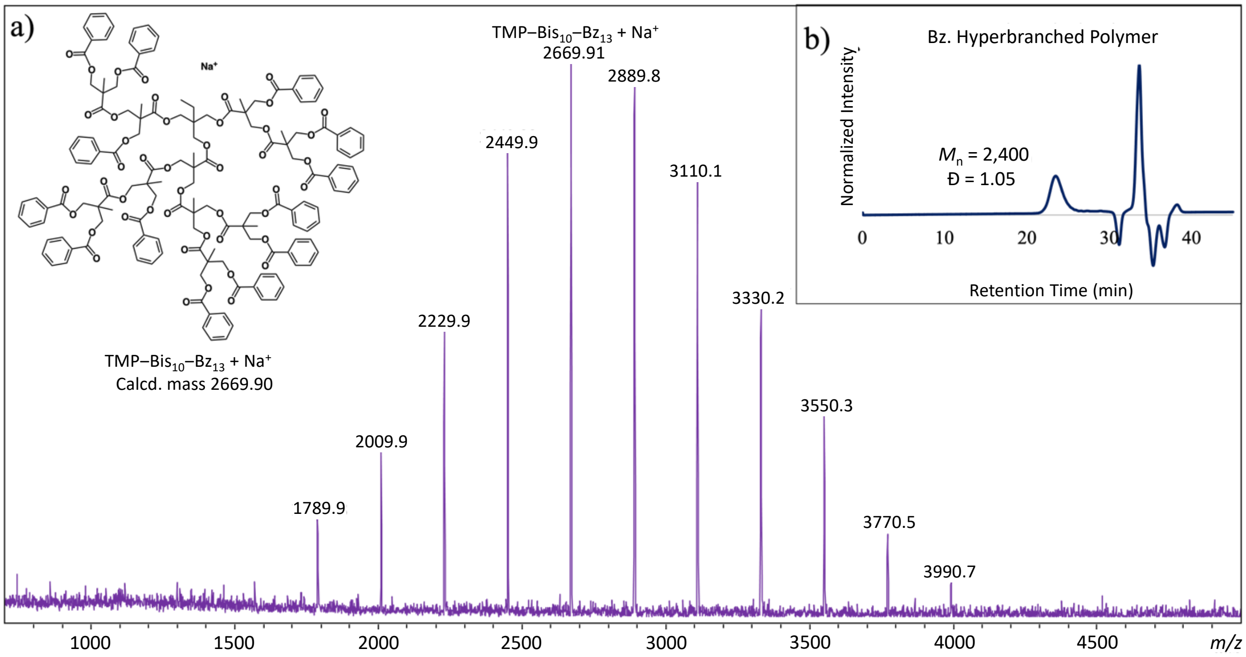

Figure S6. a) MALDI-ToF MS spectrum of TMP-Bis$_n$-Bz$_{n+3}$ hyperbranched polymer with structure and b) GPC analysis of the hyperbranched polymers (M_n = 2,400; Đ = 1.05). All masses or *m/z* ratios marked correspond to monoisotopic values.

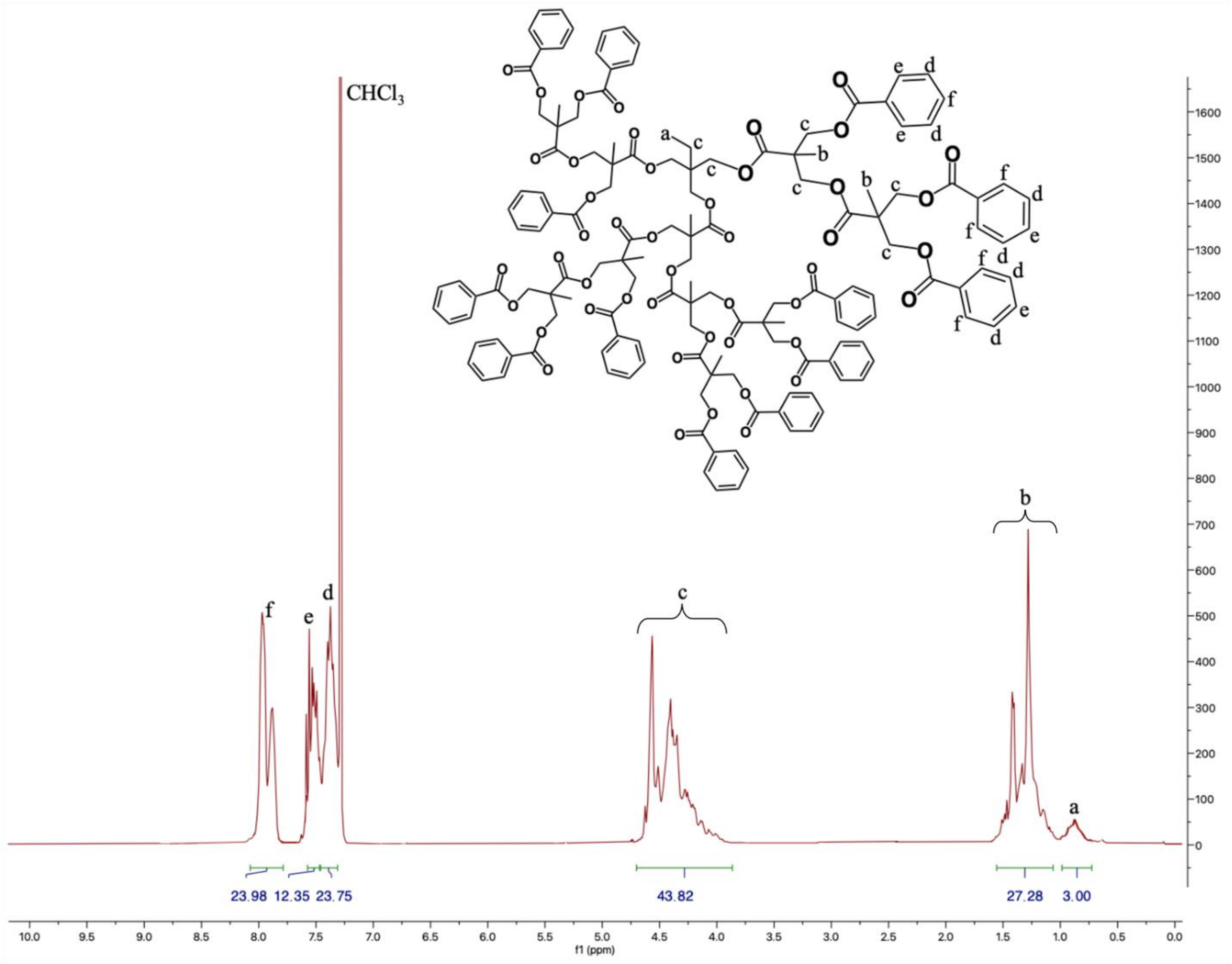

Figure S7. ^{1}H NMR spectrum of TMP-Bis$_{n}$-Bz$_{n+3}$ hyperbranched polymer with peaks observed labeled in the structure.

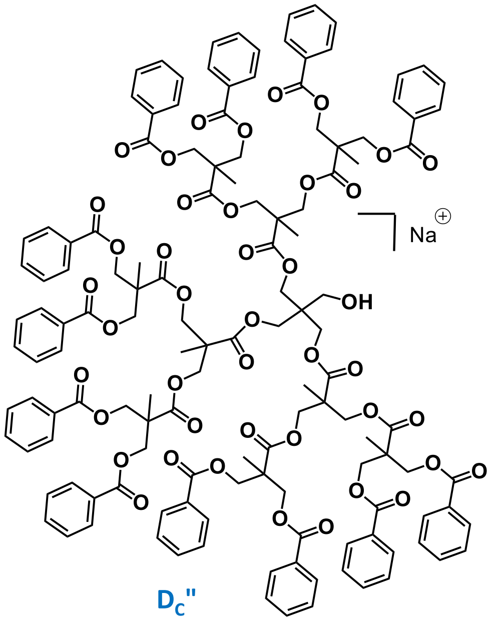

D_C''
$C_{134}H_{132}O_{43}Na^+$
Loss of $C_{43}H_{40}O_{13}$
m/z 2451.8

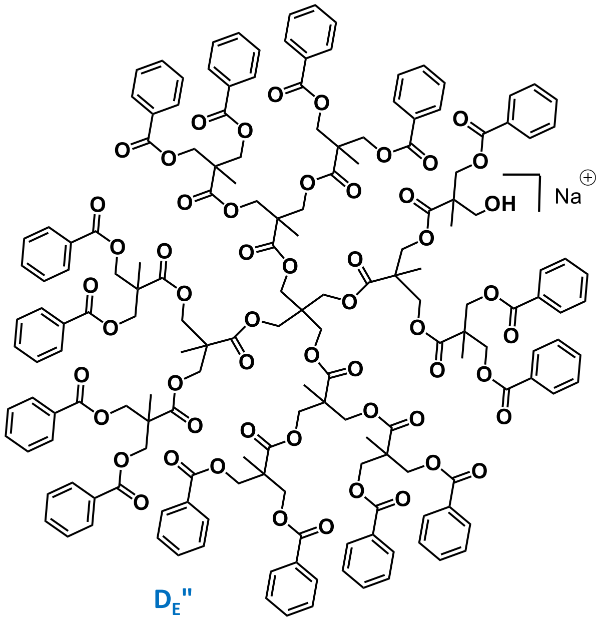

D_E''
$C_{170}H_{168}O_{55}Na^+$
Loss of C_7H_4O
m/z 3112.2

Figure S8. Fragments D_C'' and D_E'' in the MS/MS spectrum of sodiated Tetra[G2]Bz_{16} (cf. Figure 1).

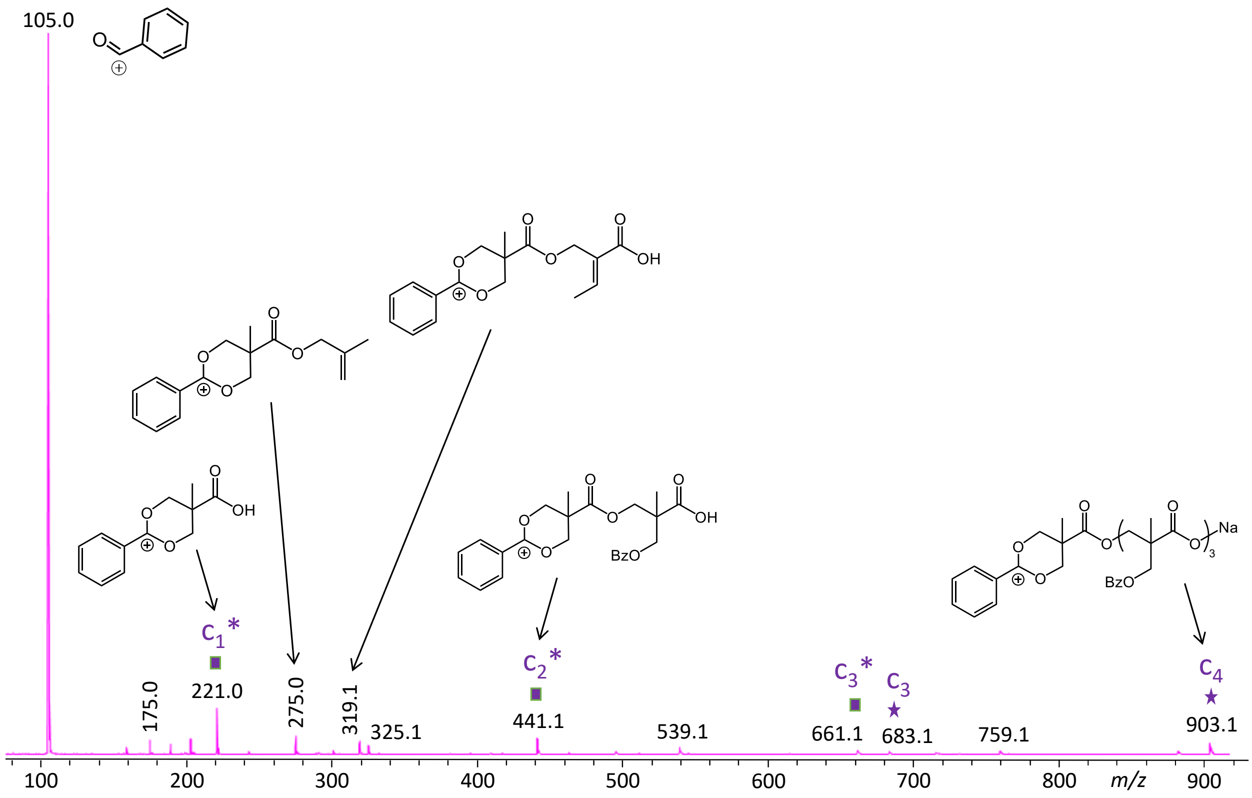

Figure S9. Low mass region of the MALDI-ToF MS/MS spectrum of sodiated PBBM 14-mer (*m/z* 3184.1). Select fragment structures are shown on top of the corresponding peaks. The ions at *m/z* 539.1 and 759.1 are longer homologs of *m/z* 319.1 (they contain 1-2 additional repeat units, respectively). The intensity of *m/z* 325.1 (relative to the benzoyl cation base peak) is <1% vs. ~18% in the MS/MS spectrum of sodiated Tetra[G2]Bz_{16} (cf. Figure 1).

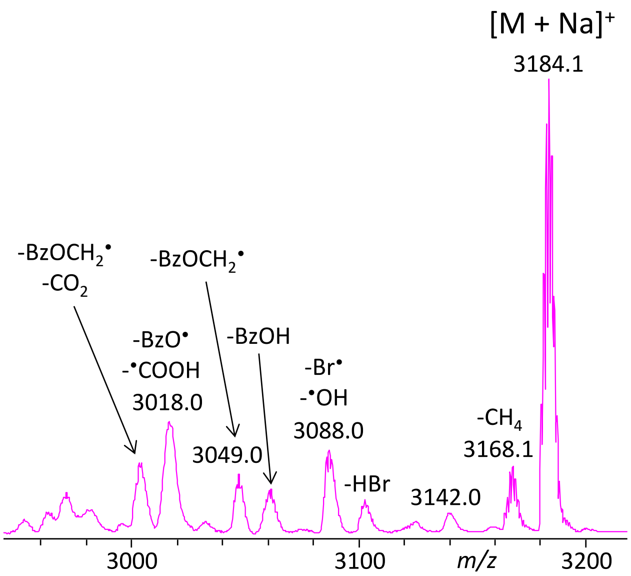

Figure S10. High mass region of the MALDI-ToF MS/MS spectrum of sodiated PBBM 14-mer (*m/z* 3184.1). The ions observed in this region correspond to fragments generated by the loss(es) of radicals or small molecules, as marked on top of the corresponding peaks.
